# Efficacy and safety of oral Chinese patent medicines in the treatment of coronary heart disease combined with hyperlipidemia: a systematic review and network meta-analysis of 78 trials

**DOI:** 10.1186/s13020-023-00866-x

**Published:** 2023-12-13

**Authors:** Zhenyu Yang, Jixin Li, Bogeng Zhou, Xuan Ji, Jianying Yuan, Junchen Yan, Xilei Nan, Dandan Guo

**Affiliations:** 1 Heilongjiang University of Traditional Chinese Medicine, Heilongjiang, 150040 China; 2 Xiyuan Hospital, China Academy of Chinese Medical Sciences, Beijing, 100091 China; 3 The Second Affiliated Hospital of Heilongjiang University of Traditional Chinese Medicine, Heilongjiang, 150001 China

**Keywords:** Chinese patent medicine, Coronary heart disease, Hyperlipidemia, Network meta-analysis, Randomized controlled trial, R 4.2.1

## Abstract

**Aim of the study:**

To evaluate the clinical efficacy and safety of commonly used oral Chinese patent medicines for the treatment of coronary heart disease combined with hyperlipidemia in clinical practice through a network meta-analysis.

**Materials and methods:**

PubMed, Embase, Cochrane Library, Web of Science, Wanfang, VIP, SinoMed, and CNKI databases were searched for all published randomized controlled trials (RCTs) on the treatment of coronary heart disease combined with hyperlipidemia using Chinese patent medicines. NoteExpress software was used to screen the literature obtained from the databases according to the inclusion and exclusion criteria. The Cochrane risk of bias assessment tool was used to evaluate the quality of the included studies. A network meta-analysis was performed using R 4.2.1. Subgroup analyses of outcome indicators were made based on conventional treatment (CT) methods. The incidence of adverse events in the included RCTs was statistically analyzed. A funnel plot was drawn using RevMan 5.4.1 software for the assessment of bias in the total clinical effectiveness rate. Finally, the quality of evidence for interventions with statistically significant differences was evaluated using the GRADE system.

**Results:**

A total of 78 RCTs were included, involving 7,955 cases and 8 types of Chinese patent medicines, which were Tongxinluo Capsule, Naoxintong Capsule, Compound Danshen Dripping Pill, Shexiangbaoxin Pill, Songling Xuemaikang Capsule, Xuezhikang Capsule, Yindan Xinnaotong Capsule, and Zhibitai Capsule. A total of 24 RCTs reported the incidence of adverse events, but no statistically significant difference in the incidence of adverse events was found between the experimental and control groups in each study (P > 0.05). There was no obvious publication bias in all studies, but the overall quality of evidence in the included RCTs was low. Comparison of different intervention measures showed that Naoxintong Capsule + CT improved the cardiac index and cardiac output, and lowered the low-density lipoprotein cholesterol and total cholesterol levels. Tongxinluo Capsule + CT raised high-density lipoprotein cholesterol levels and reduced triglyceride levels. Xuezhikang Capsule + CT improved the total clinical effectiveness rate. Subgroup analyses showed that differences in CT did not cause heterogeneity in the results.

**Conclusion:**

Compared with the use of CT alone, the combined use of Chinese patent medicines with CT can effectively improve the symptoms in patients with both coronary heart disease and hyperlipidemia.

**Supplementary Information:**

The online version contains supplementary material available at 10.1186/s13020-023-00866-x.

## Background

According to the World Health Organization (WHO), approximately 17.9 million people die from cardiovascular disease each year worldwide. This disease is the biggest threat to human health, with over 500 million patients globally. Coronary heart disease (CHD) is caused by various factors, and it can lead to atherosclerosis (AS), the narrowing of the inner diameter of blood vessels, and ultimately the deformation and even death of myocardial cells [1]. AS is a chronic disease that occurs in the walls of large- and medium-sized arteries [2], and the main cause of its formation is the accumulation of lipids and complex carbohydrates in the vascular endothelium [3]. Therefore, hyperlipidemia (HLP) is one of the independent risk factors for CHD [4]. Patients with both CHD and HLP are at high risk of cardiovascular disease. It is urgent to develop effective intervention measures to improve the symptoms in these patients.

Currently, the treatment methods for CHD combined with HLP mainly focus on reducing myocardial oxygen consumption [5] and improving myocardial blood circulation [6] while lowering blood lipid levels. Statins can stabilize vascular plaques, improve the function of endothelial cells, and most importantly, lower blood lipid levels by blocking the metabolic pathway of intracellular hydroxymethylglutaryl-coenzyme A and inhibiting cholesterol synthesis [7]. However, studies have shown that long-term use of statins can lead to abnormal liver and kidney functions, damage to nerves, muscles, and cardiovascular systems, and even death [8].

Chinese patent medicines are widely used in the treatment of CHD combined with HLP because of their stable nature, unique curative effects, relatively low toxic side effects, and ability to intervene in disease progression through different pathways and targets. However, dozens of commonly used Chinese patent medicines have not been systematically evaluated and analyzed, so there is a lack of evidence for their application in clinical practice. A network meta-analysis of the efficacy and safety of published randomized controlled trials (RCTs) on the use of Chinese patent medicines for the treatment of CHD combined with HLP was conducted in the present study. The study results provide an evidence-based medical basis for the clinical treatment of CHD combined with HLP.

## Methods

The present study was reported in strict accordance with the reporting standards outlined in the PRISMA extension statement [9] and has been registered with PROSPERO, with registration number being CRD42023411553.

### Inclusion and exclusion criteria

The criteria for including and excluding studies were developed strictly following the PICOS principles.

#### Participants

Patients with CHD and HLP met the diagnostic criteria stipulated in (1) *U.S. Guidelines for the Diagnosis and Treatment of Coronary Artery Disease 2004* [10], (2) Chinese Society of Cardiovascular Diseases Guidelines for the *Diagnosis and Treatment of Coronary Heart Disease* 2007 [11], (3) *Primary Care Guidelines for Stable Coronary Heart Disease 2020* [12], (4) *Chinese Guidelines for the Prevention and Treatment of Adult Dyslipidemia (2016 Revised Edition)* [13], etc. Patients were able to communicate and voluntarily signed the informed consent form. Patients were of different races, genders and ages.

#### Exclusion

Patients (1) with other vital organ diseases, such as hepatic or renal insufficiency, (2) with other serious cardiovascular diseases such as CHD and heart valve disease, (3) with drug allergy or a history of drug allergy, (4) with malignancy, (5) with serious mental illness, (6) during pregnancy and lactation, and (7) with other diseases were excluded.

#### Interventions and comparisons

Patients in the control group were treated with conventional clinical medications (e.g., trimetazidine, atorvastatin, lovastatin, etc.), and their diet structure was adjusted. Besides the conventional clinical medications, patients in the experimental group were also given oral Chinese patent medicines that had obtained approval from the drug regulatory authority. The dosage of Chinese patent medicines was in accordance with the requirements in the drug instruction, and the duration of treatment was unlimited. No other Chinese medical treatment measures (e.g., herbal soup, acupuncture, tui-na, etc.) were adopted in the experimental group.

#### Outcome indicators

(1) cardiac index (CI); (2) cardiac output (CO); (3) high-density lipoprotein cholesterol (HDL-C) level; (4) triglyceride (TG) level; (5) low-density lipoprotein cholesterol (LDL-C) level; (6) total cholesterol (TC) level; (7) total clinical effectiveness rate: (number of effective or apparently effective persons)/total number of persons; (8) incidence of adverse events (AEs): (number of people with AEs)/(total number of people).

#### Study design

RCTs with no restrictions on allocation concealment and blinding and language restrictions to Chinese and English.

### Literature search

Two researchers used two computers to search PubMed, Embase, Cochrane Library, Web of Science, Wanfang, VIP, SinoMed, and CNKI databases for all published RCTs on Chinese patent medicines for the treatment of CHD combined with HLP. The RCTs should be published during the period from the creation of the database to February 1, 2023. In addition, references mentioned in the included studies were supplemented. A combination of subject terms and free words was used for the search. The strategy adopted to search the Pubmed was taken as an example (Table 1).

**Table 1:** PubMed search strategy

NO.	
#1	Coronary Disease [MeSH Terms]
#2	Coronary Diseases [Title/Abstract]
#3	Disease, Coronary [Title/Abstract]
#4	Diseases, Coronary [Title/Abstract]
#5	Coronary Heart Disease [Title/Abstract]
#6	Coronary Heart Diseases [Title/Abstract]
#7	Disease, Coronary Heart [Title/Abstract]
#8	Diseases, Coronary Heart [Title/Abstract]
#9	Heart Disease, Coronary [Title/Abstract]
#10	Heart Diseases, Coronary [Title/Abstract]
#11	#1 OR #2 OR #3 OR #4 OR #5 OR #6 OR #7 OR #8 OR #9 OR #10
#12	Hyperlipidemias [MeSH Terms]
#13	Hyperlipemia [Title/Abstract]
#14	Hyperlipemias [Title/Abstract]
#15	Hyperlipidemia [Title/Abstract]
#16	Lipidemia [Title/Abstract]
#17	Lipidemias [Title/Abstract]
#18	Lipemia [Title/Abstract]
#19	Lipemias [Title/Abstract]
#20	#12 OR #13 OR #14 OR #15 OR #16 OR #17 OR #18 OR #19
#21	Chinese Patent Drugs [MeSH Terms]
#22	Chinese patent medicine [Title/Abstract]
#23	Chinese medicine [Title/Abstract]
#24	Chinese herbal medicine [Title/Abstract]
#25	Chinese Patent Drug [Title/Abstract]
#26	pill [Title/Abstract]
#27	capsule [Title/Abstract]
#28	Oral liquid [Title/Abstract]
#29	Prescription preparation [Title/Abstract]
#30	ointment [Title/Abstract]
#31	#21 OR #22 OR #23 OR #24 OR #25 OR #26 OR #27 OR #28 OR #29 OR #30
#32	randomized controlled tria [Title/Abstract]
#33	#11 AND #20 AND #31 AND #32

### Exclusion criteria

We excluded (1) non-RCTs such as reviews, conference reports, empirical summaries, theoretical explorations, treatment guidelines, etc., (2) articles with incomplete information, obvious errors in data, or academic misconduct, (3) studies using other TCM treatment methods, (4) duplicates, (5) papers concerning animal experiments and cellular experiments, (6) research with less than 30 patients included in the experimental group or in the control group, and (7) studies with more than 2 groups. In addition, (8) any Chinese patent medicine that was discussed by less than 3 studies was also excluded in the analysis of the present study.

### Literature management

NoteExpress software was used to screen the studies obtained from the databases. After duplicates were removed, the abstracts and texts of the remaining articles were further read, and articles meeting the inclusion criteria were finally included. The process was performed back-to-back by two researchers using two computers, with a third researcher extracting and comparing the results of the screening. If there was disagreement, our research team discussed it and made the final decision.

### Statistics on the basic characteristics of the included literature

Information that was extracted from the final included studies included the article authors, basic information of patients, the total sample size, interventions, duration of treatment, and outcome indicators.

### Literature quality assessment

Two evaluators, using two computers positioned back-to-back, conducted a rigorous quality assessment of the studies according to the Cochrane risk of bias assessment tool. The evaluation focused on seven aspects of each study, including whether a random allocation method was used, whether the allocation scheme was concealed, whether blinding was implemented in participants and implementers, whether blinding was implemented during outcome assessment, whether data were complete, whether there was selective reporting, and whether there were other sources of the risk of bias. The studies were rated at low risk of bias, high risk of bias, or unclear risk of bias. The evaluation results were extracted and compared, and discrepancies were discussed by our research team to determine the final result.

### Statistical analysis

The included data were processed using R 4.2.1 installed with the "BUGSnet" package and the "Just Another Gibbs Sampler (JAGS)" generalized linear models [14]. Specifically, (1) the "n ma.model()" function was used to parameterize the effect model data, evaluate the fitness of each model, and identify potential outliers. It output the "leverage plot" and the "deviance information criterion (DIC)". (2) The "net.plot()" function was used for descriptive statistics analysis of the obtained evidence and to draw a "network diagram of evidence". In addition, for graphs that had a closed-loop structure, an "inconsistency test" was performed. (3) The "n ma.forest()" function was used to make a meta-analysis of the interventions and underlying treatment methods in each study. It output a "forest plot". (4) The "n ma.league()" function was used to evaluate the relative effects of the interventions in each study and draw a "heat map ". (5) The "n ma.rank()" function was used to rank the cumulative probabilities of the interventions in each study, calculate the "surface under the cumulative ranking (SUCRA)” and pot the "ranking graph". Subgroup analyses were performed for any significant differences between groups, and sensitivity analyses were performed for factors that caused bias in the results.

### Security analysis

The incidence of AEs in the included RCTs was calculated, and the difference in the incidence of AEs between the experimental and control groups in each study was examined to see if there was any statistical significance.

### Risk of publication bias

A funnel plot was drawn using RevMan 5.4.1 software for the assessment of bias in the total clinical effectiveness rate.

### GRADE for the evaluation of the quality of evidence

The quality of evidence for interventions with statistically significant differences was evaluated using the GRADE system. The evaluation included 5 factors for downgrading evidence, which were Research limitations inconsistency, indirectness, imprecision, and publication bias. Finally, the quality of evidence was awarded 4 grades of high, moderate, low, and very low.

## Results

### Basic characteristics of the included literature

**Fig. 1:**
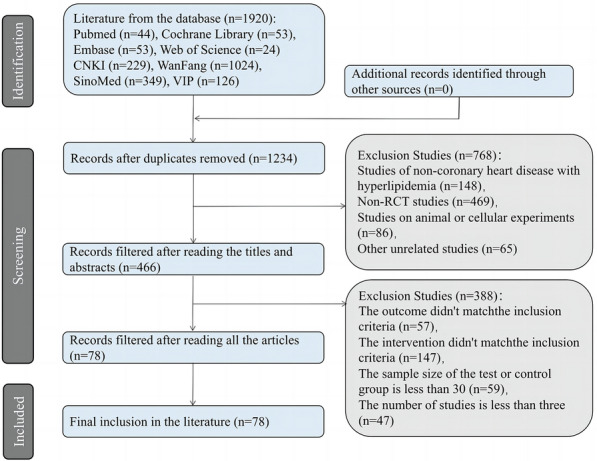
Flow diagram depicting the study screening process

A preliminary search of the databases yielded a total of 1920 relevant studies, and 1234 studies remained after duplicates were removed. Then these articles were screened strictly using NoteExpress according to the inclusion and exclusion criteria, and 78 studies [15–92] were finally included. The flow chart of the screening process is shown in Fig. 1. A total of 7955 cases were included in all studies, with 4019 cases in the experimental group and 3936 cases in the control group. The sample sizes of the studies ranged from 60 to 200 cases, with a mean of 102 cases. Eight Chinese patent medicines were investigated in the studies, including Compound Danshen Dripping Pill (DSDW) [15–41], Naoxintong Capsule (NXT) [42–55], Shexiangbaoxin Pill (SXBXW) [56–61], Songling Xuemaikang Capsule (XMK) [62–64], Tongxinluo Capsule (TXL) [65–80], Xuezhikang Capsule (XZK) [81–85], Yindan Xinnaotong Capsule (YDXNT) [86–89], and Zhibitai Capsule (ZBT) [90–92]. The control group mainly received conventional treatment (CT), while the experimental group was given both conventional treatment and traditional Chinese medicines. Information from the finally included studies was collected, and their basic characteristics are presented in Additional file 1.

### Quality evaluation of the included literature

**Fig. 2:**
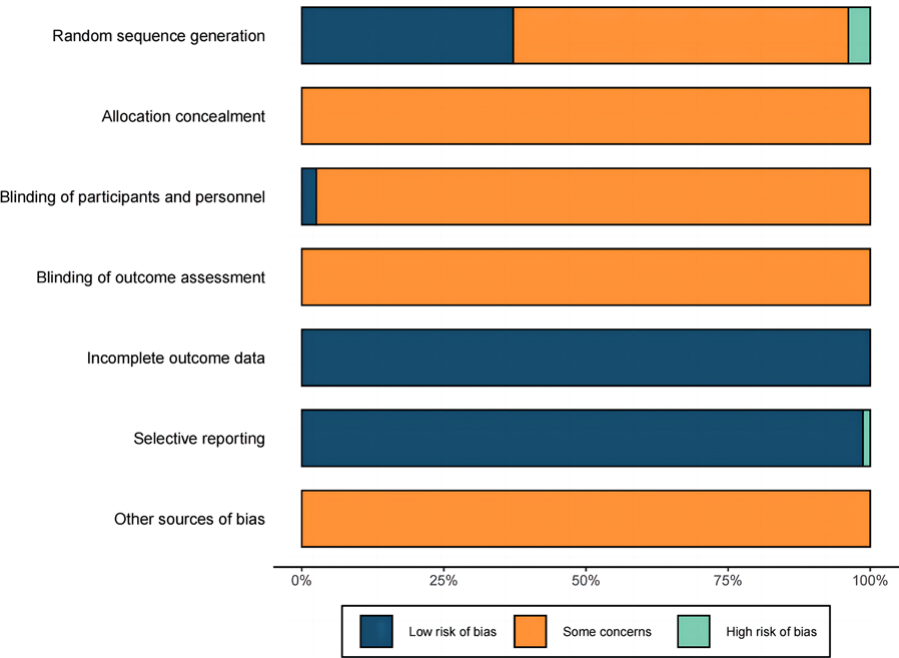
Quality evaluation chart of the included literature

Specific methods of random assignment were reported in 32 [15, 17, 18, 20, 21, 25, 26, 28–32, 34–37, 52, 55–57, 59–61, 63, 70, 74–77, 79, 88, 89] of the 78 RCTs. Of these 32 reports, 29 studies [15, 17, 20, 21, 25, 26, 28–32, 34–37, 52, 55–57, 59–61, 63, 70, 74, 77, 79, 88, 89] used the random number table method and the remaining 3 papers [18, 75, 76] used an incorrect randomization method (ranking patients according to their order of hospitalization). All studies did not specify whether the assignment scheme was concealed. In two studies [33, 80], both subjects and principal investigators were blinded, and blinding was not broken. All studies did not mention whether blinding was applied to outcome assessors. The outcome information was complete in all studies. Only 1 study [38] had selective reporting of outcomes, and it was unclear whether there were other biases. The quality evaluation results of the included literature are shown in Fig. 2.

### Choice of the effect model

Random-effects models and fixed-effects models were selected based on the calculated DIC values for the analysis of seven outcome indicators in the present study. The effect models with lower DIC values were selected for analysis [14], and the results are shown in Additional file 2.

### Cardiac index

#### Evidence network

**Fig. 3:**
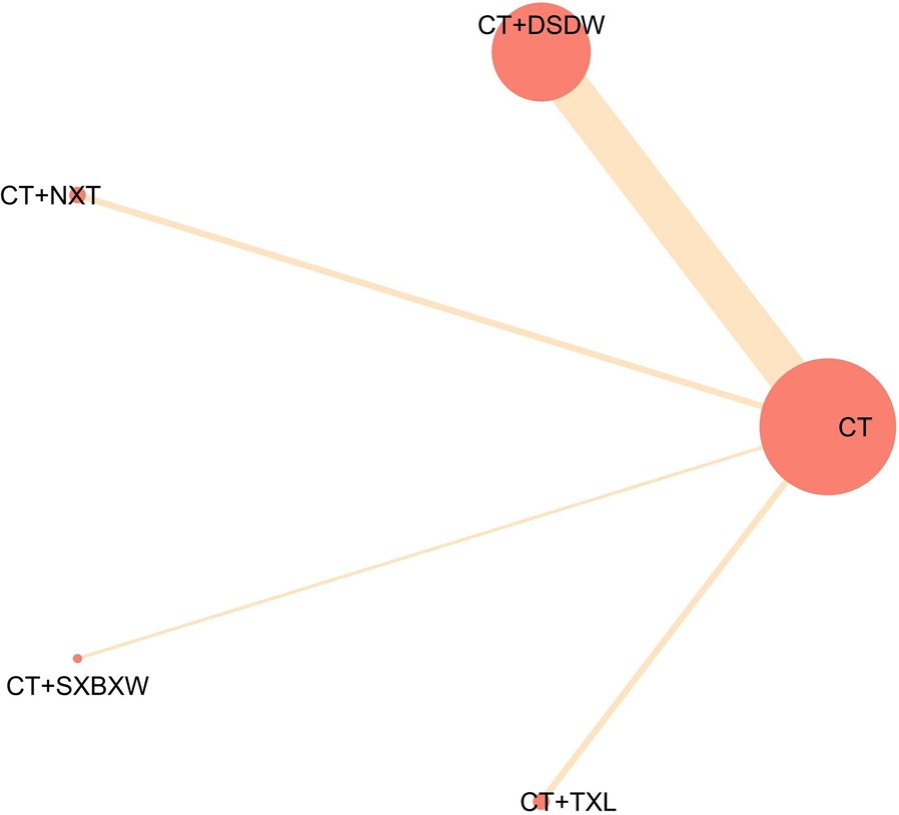
Evidence network map of CI

The statistical analysis of CI as an outcome measure was made in 18 RCTs [15, 17, 19, 20, 26, 27, 29, 30, 32, 35, 37–39, 42, 51, 60, 67, 70], covering 4 Chinese patent medicines and 2144 patients. These studies directly compared different intervention measures without forming a closed loop. The network relationship among intervention measures is shown in Fig. 3. It is evident that each node represents an intervention measure, and its size is positively correlated with the number of studies, as is the width of the connections between nodes. Most of these RCTs (13 studies) were related to DSDW.

#### Estimation of the relative effects of each intervention

**Fig. 4:**
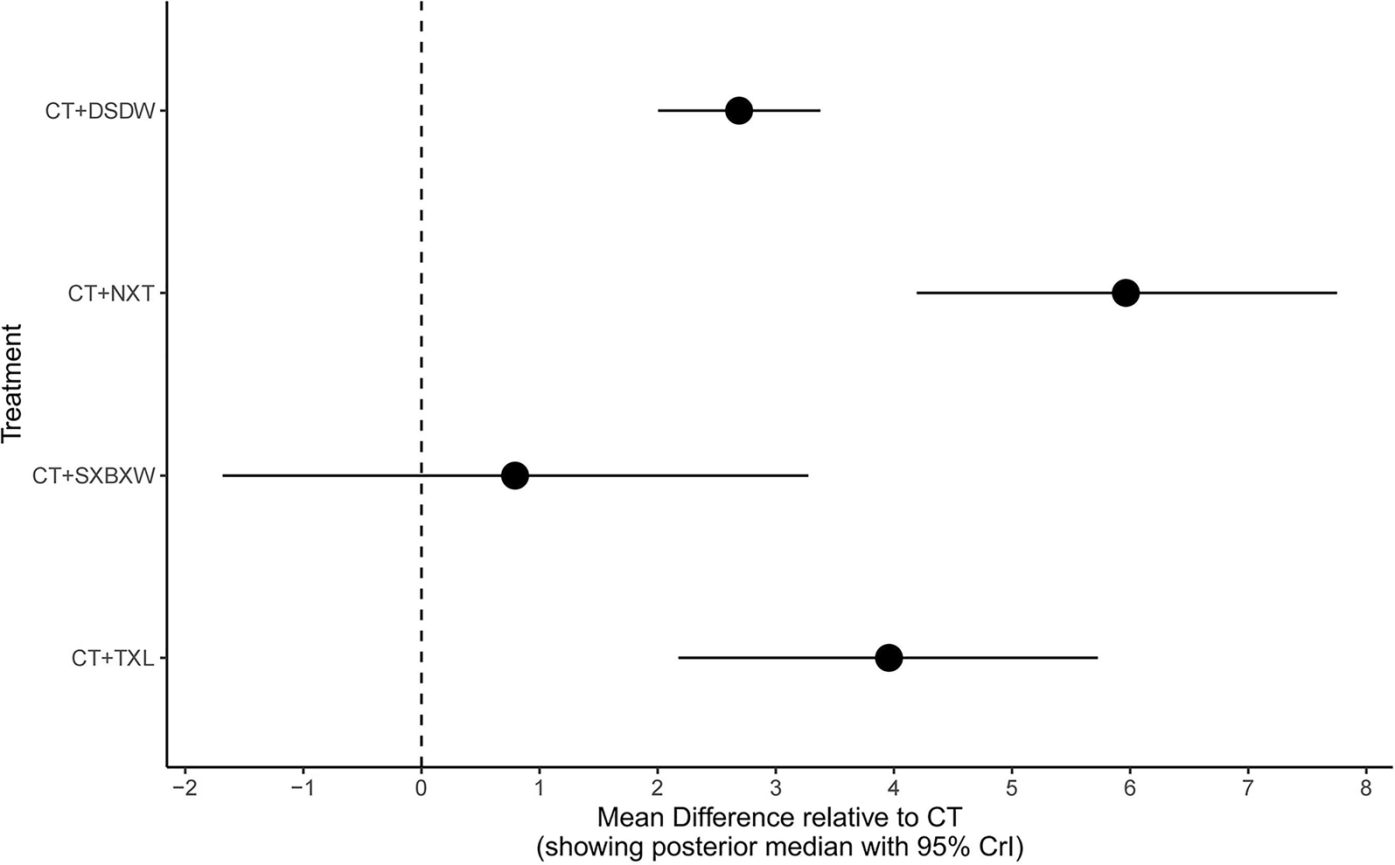
Direct comparison forest map of CI [MD(95%CI)]

**Fig. 5:**
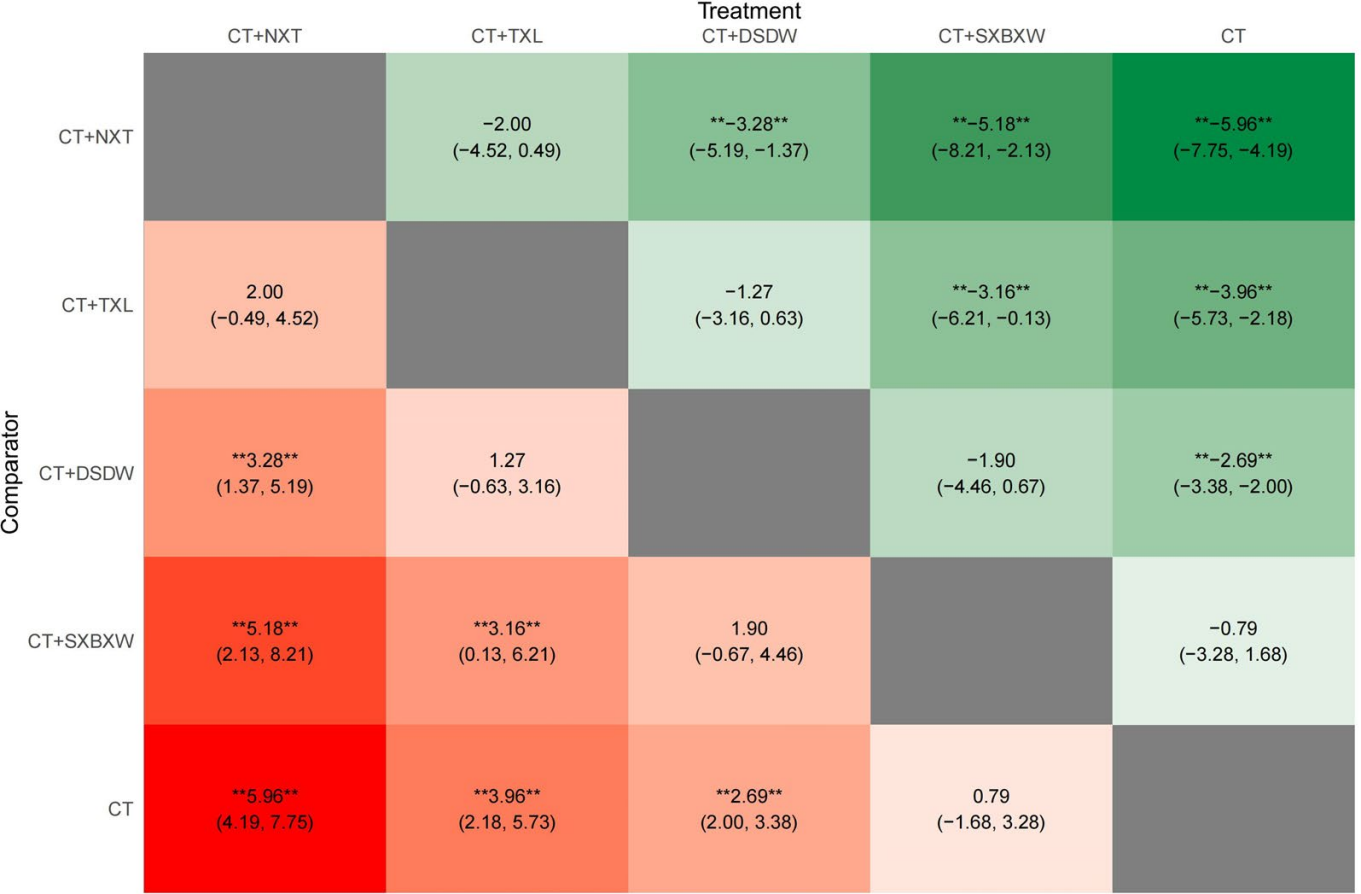
Network Meta-analysis heat map of CI [MD(95%CI)]

Direct comparisons were made between intervention measures adopted in the studies (Fig. 4). A random-effects model was selected based on the DIC value, and the results were summarized using a forest plot. Compared with CT, DSDW + CT, NXT + CT, and TXL + CT significantly improved CI (all P < 0.05). However, no statistically significant difference in CI improvement was observed between SXBXW + CT and CT (P > 0.05). The relative effects of the interventions were demonstrated by a heat map (Fig. 5). NXT + CT were superior to SXBXW + CT and DSDW + CT in improving CI (both P < 0.05). TXL + CT showed better effect in improving CI than SXBXW + CT (P < 0.05). There was no statistically significant difference in CI improvement among the remaining interventions in the experimental groups (all P > 0.05).

#### Comparison of rankings across interventions

**Fig. 6:**
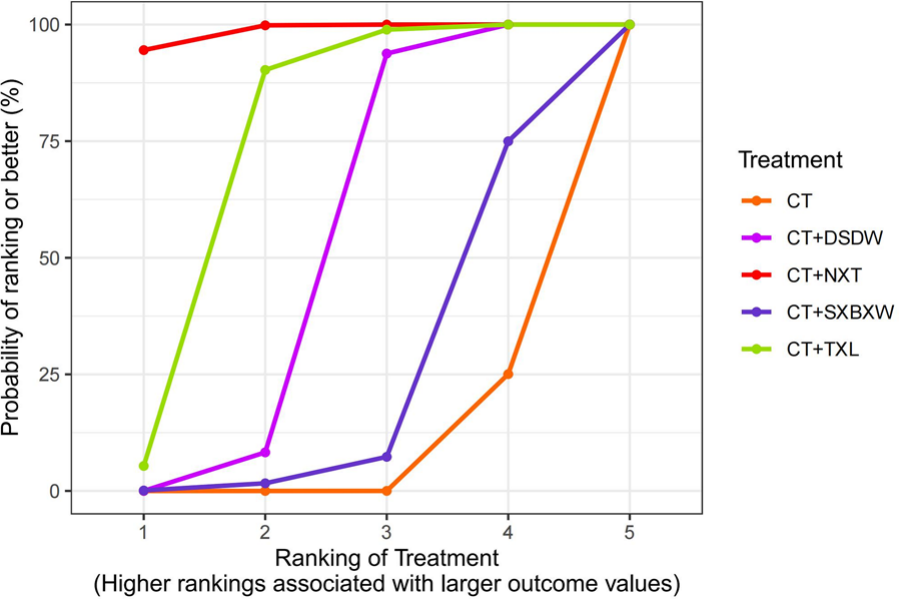
Probability ranking curves of the degree of improvement of CI [MD(95%CI)]

The probability ranking curve of different intervention measures is plotted in Fig. 6. It is evident that SUCRA values are positively correlated with the CI-improving effect of intervention measures. Finally, the rankings of the intervention measures by the CI-improving effect were: NXT + CT (98.60%) > TXL + CT (73.65%) > DSDW + CT (50.57%) > SXBXW + CT (20.94%) > CT (6.24%).

#### Sensitivity analysis

Only one study [60] on SXBXW was conducted, so it was excluded. SUCRA values showed that NXT + CT had the highest sensitivity (99.08%), followed by TXL + CT (74.75%), DSDW + CT (51.28%) and CT (7.06%) successively. This ranking result was not significantly different from the results achieved by previous studies. It proves that the results of this network meta-analysis are reliable.

### Cardiac output

#### Evidence network

19 RCTs [15, 17, 19, 20, 25–27, 29, 30, 32, 35, 37–39, 42, 51, 60, 67, 70] reported the statistical analysis results of CO as an outcome indicator, involving 4 Chinese patent medicines and 2310 patients. These studies directly compared different intervention measures without forming a closed loop. The network relationship among intervention measures is illustrated in Additional file 3. It is obvious that most studies (14 out of 19 RCTs) were related to DSDW.

#### Estimation of the relative effects of each intervention

DSDW + CT, NXT + CT, and TXL + CT significantly improved CO, compared with CT (all P < 0.05) (Additional file 4). Nevertheless, no statistically significant difference in CO improvement was found between SXBXW + CT and CT (P > 0.05). It can be seen from Additional file 5 that there was no statistically significant difference in CO improvement among the experimental groups (all P > 0.05).

#### Comparison of rankings across interventions

Additional file 6 shows the rankings of different interventions by the CO-improving effect: NXT + CT (90.75%) > TXL + CT (65.82%) > DSDW + CT (60.98%) > SXBXW + CT (29.05%) > CT (3.39%).

#### Sensitivity analysis

Only one study [60] on SXBXW was conducted, so it was excluded. The SUCRA values showed that NXT + CT (91.93%) had the highest sensitivity, followed by TXL + CT (67.64%), DSDW + CT (61.88%) and CT (4.61%) successively. This ranking result was not significantly different from the results obtained by previous investigations. Thus, the results of this network meta-analysis are reliable.

### High-density lipoprotein cholesterol level

#### Evidence network

In 57 RCTs [15, 17, 18, 23, 25, 26, 28, 30, 32–34, 36, 37, 39, 42–50, 52–55, 58–60, 62–67, 69–74, 76, 78–81, 83–92], totaling 5812 patients, a statistical analysis of the HDL-C level as an outcome measure was made, with 8 Chinese patent medicines discussed. These studies directly compared different intervention measures without forming a closed loop. The network relationship among intervention measures is described in Additional file 7. It is evident that most studies (14 out of 57 RCTs) were related to DSDW.

#### Estimation of the relative effects of each intervention

DSDW + CT, NXT + CT, SXBXW + CT, and TXL + CT significantly improved the HDL-C level, compared with CT (all P < 0.05) (Additional file 8). However, CT was not significantly different from SXBXW + CT, XZK + CT, YDXNT + CT, and ZBT + CT in improving the HDL-C level (all P > 0.05). As shown in Additional file 9 TXL + CT and NXT + CT were superior to YDXNT + CT in improving the HDL-C level (both P < 0.05). In addition, there was no statistically significant difference in the effect of improving the HDL-C level among the interventions in the experimental groups (all P > 0.05).

#### Comparison of rankings across interventions

Additional file 10 provides the rankings of different interventions by the effect of improving the HDL-C level: TXL + CT (83.15%) > NXT + CT (75.59%) > SXBXW + CT (70.98%) > DSDW + CT (57.87%) > XZK + CT (49.38%) > XMK + CT (46.68%) > ZBT + CT (39.60%) > YDXNT + CT (15.46%) > CT (11.28%).

#### Sensitivity analysis

Only 3 studies [62–64, 90–92] focused on XMK and ZBT, so they were excluded. The SUCRA values showed that TXL + CT (84.24%) has the highest sensitivity, followed by NXT + CT (78.07%), SXBXW + CT (72.13%), DSDW + CT (58.91%), XZK + CT (51.318%), YDXNT + CT (17.69%) and CT (13.02%) successively. This ranking result was not significantly different from the results achieved by previous research. It demonstrates that the results of this network meta-analysis are reliable.

### Triglyceride level

#### Evidence network

In 70 RCTs [15–21, 23, 25, 27, 29–33, 35, 36, 38–72, 74–81, 83–92] involving 7181 patients, a statistical analysis of the TG level as an outcome measure was made and 8 Chinese patent medicines were investigated. These studies directly compared different intervention measures without forming a closed loop. The network relationship among intervention measures is described in Additional file 11. It is evident that 21 out of 70 RCTs investigated DSDW, making DSDW the most investigated medicine among the 8 Chinese patent medicines.

#### Estimation of the relative effects of each intervention

It can be seen from Additional file 12 that DSDW + CT, NXT + CT, SXBXW + CT, and TXL + CT reduced the TG level more significantly than CT (all P < 0.05). However, CT was not statistically significantly different from XMK + CT, XZK + CT, YDXNT + CT, and ZBT + CT in TG reduction (all P > 0.05). According to Additional file 13, TXL + CT was superior to DSDW + CT, XMK + CT, XZK + CT, YDXNT + CT and ZBT + CT in reducing the TG level (P < 0.05). NXT + CT reduced the TG more significantly than XMK + CT, XZK + CT, YDXNT + CT and ZBT + CT (all P < 0.05). DSDW + CT exhibited better effect of reducing the TG level than YDXNT + CT and ZBT + CT (both P < 0.05). In addition, the remaining interventions in experimental groups did not differ from each other significantly in TG reduction (P > 0.05).

#### Comparison of rankings across interventions

Additional file 14 ranks different interventions by TG reduction: TXL + CT (94.99%) > NXT + CT (90.21%) > DSDW + CT (68.63%) > SXBXW + CT (62.63%) > XMK + CT (41.04%) > XZK + CT (38.95%) > YDXNT + CT (29.91%) > ZBT + CT (18.11%) > CT (5.51%).

#### Sensitivity analysis

Only 3 studies were related to XMK and ZBT [62–64, 90–92], so they were excluded. The SUCRA values revealed that TXL + CT (96.28%) had the highest sensitivity, followed by NXT + CT (91.65%), DSDW + CT (69.77%), SXBXW + CT (63.84%), XZK + CT (40.05%), YDXNT + CT (31.22%) and CT (7.19%) successively. No significant difference was observed between this ranking result and the results of previous research, demonstrating the reliability of the results of this network meta-analysis.

### Low-density lipoprotein cholesterol level

#### Evidence network

The statistical analysis of the LDL-C level as an outcome measure was made in 67 RCTs, involving 6913 patients, with 8 Chinese patent medicines discussed. These studies directly compared different intervention measures without forming a closed loop. Additional file 15 illustrates the network relationship among intervention measures. It is obvious that most studies (25 out of 67 RCTs) focused on DSDW.

#### Estimation of the relative effects of each intervention

DSDW + CT, NXT + CT, XZK + CT, and TXL + CT reduced the LDL-C level more significantly than CT (all P < 0.05) (Additional file 16). However, CT was not statistically significantly different from XMK + CT, SXBXW + CT, YDXNT + CT, and ZBT + CT in lowering the LDL-C level (all P > 0.05). As shown in Additional file 17, NXT + CT performed better than DSDW + CT, XMK + CT, XZK + CT, YDXNT + CT, ZBT + CT and SXBXW + CT in LDL-C reduction (all P < 0.05). TXL + CT was superior to XMK + CT, ZBT + CT, YDXNT + CT and SXBXW + CT in reducing the LDL-C level (all P < 0.05). DSDW + CT lowered the LDL-C level more significantly than SXBXW + CT, YDXNT + CT and ZBT + CT (all P < 0.05). In addition, the remaining interventions in experimental groups did not differ statistically significant in LDL-C reduction (P > 0.05).

#### Comparison of rankings across interventions

The rankings of different interventions by LDL-C reduction were NXT + CT (97.97%) > TXL + CT (85.01%) > DSDW + CT (77.44%) > XZK + CT (52.80%) > XMK + CT (40.49%) > YDXNT + CT (32.98%) > ZBT + CT (32.85%) > SXBXW + CT (22.78%) > CT (7.66%) (Additional file 18).

#### Sensitivity analysis

Only 3 papers investigated XMK and ZBT [62–64, 90–92], so they were excluded. Based on the SUCRA values, NXT + CT (98.35%) had the highest sensitivity, followed by TXL + CT (88.11%), DSDW + CT (79.06%), XZK + CT (54.82%), YDXNT + CT (35.27%), SXBXW + CT (24.61%), and CT (9.89%) successively. This ranking result was not significantly different from the results of previous studies, indicating that the results of this network meta-analysis are reliable.

### Total cholesterol level

#### Evidence network

In 76 RCTs [15–21, 23–72, 74–92] totaling 7735 patients, the TC level was analyzed as an outcome indicator, and 8 Chinese patent medicines examined. These studies directly compared different intervention measures without forming a closed loop. The network relationship among intervention measures is given in Additional file 19. It can be seen from the figure that 26 RCTs were related to DSDW, which was thus the most investigated medicine.

#### Estimation of the relative effects of each intervention

Compared with CT, DSDW + CT, NXT + CT, XZK + CT, TXL + CT, XMK + CT, SXBXW + CT and YDXNT + CT reduced the TC level significantly (all P < 0.05) (Additional file 20). In contrast, no statistically significant difference was found in TC reduction between ZBT + CT and CT (P > 0.05). As shown in Additional file 21, NXT + CT performed better than TXL + CT, XMK + CT, XZK + CT, YDXNT + CT, ZBT + CT and SXBXW + CT in reducing the TC level (all P < 0.05). DSDW + CT was superior to TXL + CT, XZK + CT and ZBT + CT in TC reduction (all P < 0.05). In addition, the remaining interventions in experimental groups did not differ significantly from each other in TC reduction (all P > 0.05).

#### Comparison of rankings across interventions

Additional file 22 shows the rankings of different interventions by TC reduction:

NXT + CT (97.58%) > DSDW + CT (87.52%) > TXL + CT (54.12%) > SXBXW + CT (51.93%) > YDXNT + CT (50.44%) > XMK + CT (45.13%) > XZK + CT (39.92%) > ZBT + CT (21.38%) > CT (2.00%).

#### Sensitivity analysis

XMK and ZBT were analyzed only in 3 studies [62–64, 90–92], which were thus excluded. Based on the SUCRA values, the sensitivity of NXT + CT (98.74%) ranked the highest, followed by DSDW + CT (89.09%), TXL + CT (55.85%), SXBXW + CT (53.41%), YDXNT + CT (52.27%), XZK + CT (41.02%) and CT (3.68%) successively. There is no significant difference between this ranking result and the results of previous reports, proving the reliability of the results of this network meta-analysis.

### Total clinical effectiveness rate

#### Evidence network

The total clinical effectiveness rate was studied as an outcome measure in 62 RCTs [15–19, 21–28, 30–32, 34–38, 40–42, 46–48, 50–52, 54, 55, 57–63, 65, 66, 68–80, 83, 84, 86, 87, 89–92], covering 8 Chinese patent medicines and 6482 patients. These studies directly compared different intervention measures without forming a closed loop. The network relationship among intervention measures is described in Additional file 23. Among the 8 Chinese patent medicines, DSDW was the most studied (23 RCTs).

#### Estimation of the relative effects of each intervention

All interventions in the experimental groups improved the total clinical effectiveness rate more significantly than CT in the control group (all P < 0.05) (Additional file 24). According to Additional file 25, XZK + CT was superior to ZBT + CT in improving the total clinical effectiveness rate (P < 0.05). In addition, there was no statistically significant difference in the improvement of the total clinical effectiveness rate among the remaining interventions in the experimental groups (P > 0.05).

#### Comparison of rankings across interventions

Additional file 26 shows the rankings of different interventions by the effect of improving the total clinical effectiveness rate: XZK + CT (94.92%) > DSDW + CT (67.16%) > SXBXW + CT (66.27%) > TXL + CT (57.81%) > YDXNT + CT (55.45%) > NXT + CT (45.29%) > XMK + CT (40.05%) > ZBT + CT (22.99%) > CT (0.07%).

#### Sensitivity analysis

XMK and ZBT were studied only in 3 studies [62–64, 90–92], which were therefore excluded. The SUCRA values suggested that XZK + CT (97.11%) had the highest sensitivity, followed by DSDW + CT (69.25%), SXBXW + CT (68.71%), TXL + CT (59.37%), YDXNT + CT (55.45%), NXY + CT (48.02%) and CT (2.31%) successively. This ranking result was not significantly different from the results of previous research, demonstrating that the results of this network meta-analysis are reliable.

### Security analysis

The incidence of AEs was calculated in 24 RCTs [18, 25, 45–50, 53, 54, 57, 62, 64, 66, 71, 76, 78, 81–83, 85, 87, 90, 92], 13 [25, 45, 46, 48, 53, 54, 57, 62, 66, 71, 76, 87, 90] of which reported the occurrence of AEs (Additional file 27). The difference in the incidence of AEs between the experimental and control groups in each study was not statistically significant (P > 0.05).

### Subgroup analysis based on different CT methods

Among the 78 RCTs included in the present study, seven different CT measures were used, based on which subgroup analyses were made (Additional file 28). The rankings of the CT measures by the SUCRA values shown in the table were essentially in accordance with the results of the present study, indicating that the differences in CT measures have less impact on the results.

### Risk of publication bias

A funnel plot was drawn using RevMan 5.4.1 software to assess the bias in the total clinical effectiveness rate (Additional file 29). All eight types of interventions were located within the 95% CI, and the plot was symmetrical, suggesting that there is no significant publication bias in all the studies.

### Evidence quality evaluation by GRADE

The results showed that the quality of evidence for the included interventions was low [15, 17, 20, 21, 25, 26, 28–37, 52, 55–57, 59–61, 63, 70, 74, 77, 79, 80, 88, 89] or very low [16, 18, 19, 22–24, 27, 38–51, 53, 54, 58, 62, 64–69, 71–73, 75, 76, 78, 81–87, 90–92]. The overall quality of evidence was rated low because of the high risk of bias due to the fact that most studies did not mention whether blinding or allocation concealment was implemented. Moreover, the heterogeneity of the included studies and the differences in sample size led to inconsistency, imprecision, and serious indirect transmission.

## Discussion

### Summary of results

A total of 78 RCTs were included in the present study. All of the 8 interventions were correlated with 7 outcome indicators, and a horizontal comparison between different interventions was made. As indicated by all the 7 outcome indicators, the clinical efficacy of the Chinese patent medicine and CT was significantly better than that of CT alone. This result proves that the combination of Chinese patent medicines and CT can effectively treat CHD combined with HLP. It should be noted that due to the multi-target and multi-pathway characteristics of Chinese herbal components, different components may not necessarily have the same regulatory effect through the same pathway, but rather exert an overall effect [93].

Among all the RCTs, most research discussed DSDW, totaling 27. It suggests that DSDW is currently the most commonly used Chinese patent medicine for the clinical treatment of CHD combined with HLP. Comparison of different intervention measures showed that NXT + CT performed the best in improving CI and CO and lowering LDL-C and TC levels. TXL + CT raised HDL-C levels and reduced TG levels the most significantly. XZK + CT greatly improved the total clinical effectiveness rate.

### Naoxintong capsule

According to existing systematic reviews and meta-analyses [94–96], NXT has a protective effect against a variety of cardiovascular diseases.

NXT contains various compounds and has multiple targets of action. It can protect the heart through multiple pathways. Traditional Chinese medicine believes that blood stasis is an important pathological factor leading to CHD. Specifically, blood stasis stagnates in the meridians, impeding the smooth flow of Qi in the body, affecting fluid metabolism, and ultimately leading to symptoms such as edema, which further increase the burden on the heart. Therefore, promoting blood circulation, resolving blood stasis, warming yang, and promoting diuresis are the guiding principles of traditional Chinese medicine in treating CHD. NXT is composed of 16 Chinese herbal medicines, including insect medicines and blood-activating and stasis-removing medicines, with the effects of benefiting qi, activating blood, and dredging collaterals [97]. Q. Li [98] et al. searched the HIT 2.0 database for the targets of action of the drug components contained in NXT and conducted a KEGG pathway enrichment analysis. The results showed that NXT produces the effects of anti-inflammation, anti-oxidation, anti-coagulation, protecting vascular endothelial cells, and inhibiting cell apoptosis through multiple pathways. Vascular endothelial cells play an important role in maintaining the normal function of blood vessels and promoting angiogenesis [99]. They can synthesize and release plasminogen activator inhibitor-1 (PAI-1) to inhibit the activity of the plasminogen activator [100]. Li et al. [101] made UPLC-Q-TOF/MS, plasma metabolomics and network pharmacology analyses. It was found that NXT regulated the metabolism of arachidonic acid, the synthesis of steroid hormones, the synthesis of primary bile acids, and the metabolism of sphingolipids, thereby achieving the effect of promoting blood circulation and removing blood stasis.

### Tongxinluo capsule

Published meta-analyses [102–104] have confirmed that TXL, as a complementary drug, can significantly improve the effect of CT in treating CHD and reduce lipid levels.

TXL has the functions of benefiting qi, promoting blood circulation, and dredging collaterals, and is widely used in the treatment of CHD [105]. CHD is essentially an inflammatory response. The inflammatory state triggers early endothelial damage in patients with CHD and ultimately leads to the formation of atherosclerotic plaques. TXL can significantly reduce the level of inflammatory molecules in patients with CHD combined with HLP, thus alleviating the body's inflammatory responses. X. Jiang et al. have confirmed that TXL can reduce the expression of related inflammatory factors and lower blood lipid levels by inhibiting the Wnt/β-catenin pathway [106]. TXL also protects blood, blood vessels and ischemic tissue, plays an anticoagulant effect, stabilizes plaques and relieves vascular spasm. TXL has a strong protection effect on microvessels. With unique advantages, TXL has become the preferred medicine for the treatment of cardiovascular and cerebrovascular diseases [107].

### Xuezhikang capsule

XZK + CT is significantly superior to CT therapies alone in TC reduction, as confirmed by systematic reviews and meta-analyses published to date [108, 109].

XZK is extracted from the traditional Chinese medicine red yeast rice and has the effects of resolving turbidity, reducing lipids, promoting blood circulation, removing blood stasis, invigorating the stomach and promoting digestion. HLP first damages and causes the detachment of endothelial cells, leading to increased vascular permeability. Subsequently, lipoproteins enter the blood vessels and deposit on the intima of the vessel wall. Eventually, macrophages in the body tissues proliferate in response to clearance reactions and in conjunction with vascular smooth muscle cells, forming atherosclerotic plaques, which accelerate the progression of CHD. Modern pharmacological studies have confirmed that the main components of XZK, including natural compound statins, monacolins, and ergosterol, can activate the PI3K/PKB/Akt pathway to increase the activity of endothelial Nitric Oxide Synthases (eNOS), thereby improving the function of endothelial cells [110]. Moreover, XZK also inhibits the phosphorylation of c-Jun N-terminal kinase (JNK) by maintaining the activity of peroxisome proliferator-activated receptor γ (PPARγ), reduces the release of inflammatory factors, and ultimately mitigates the inflammatory response [111].

### Other Chinese patent medicines

There is multiple evidence that DSDW [112–114], SXBXW [115–117], and ZBT [118, 119] can reduce the risk of CHD to varying degrees. In conclusion, there are various oral Chinese patent medicines available for clinical use. Different Chinese patent medicines may have different therapeutic targets, and their effects on the same outcome also vary. Therefore, in clinical practice, it is important to use appropriate Chinese patent medicines based on the specific condition of each patient (Additional file 30, Additional file 31).

### Limitations of the present study

In this study, a total of eight Chinese patent medicines used for the clinical treatment of CHD combined with HLP were discussed, and their effects are reliable with few side effects. Therefore, they are expected to become the first-line drugs for the clinical treatment of this disease. However, during the process of literature review, the authors identified some limitations in the current research field. (1) Firstly, most of the included RCTs did not report whether allocation concealment or blinding was implemented, so the overall quality of evidence was assessed as low. (2) Secondly, secondary prevention of CHD recommends lowering blood lipids to the target value, but only 9 studies y [34, 47, 55, 62, 63, 71, 73, 76, 79] reported the achievement of target blood lipid levels in patients after treatment. Further research and analysis are needed for the effects of Chinese patent medicines in helping the patient to achieve target blood lipid levels. (3) Thirdly, only 59 RCTs reported diagnostic criteria. There were many diagnostic bases and the differences between diagnostic bases were insignificant. Therefore, the subgroup analysis based on the type of diagnostic criterion was not applicable due to an insufficient amount of literature included in the subgroups, and it would make it difficult to compare different treatment options. Differences in diagnostic criteria may be a source of heterogeneity in this paper because subgroup analyses were not performed based on diagnostic criteria. The results of this study were obtained by the data analysis of the included literature, and may not accurately reflect the specific effect of clinical treatment. Therefore, the drugs discussed in this study should be selected with caution for clinical application. In the future, it is necessary to conduct further multicenter, double-blind and large-sample RCTs.

### Supplementary Information

**Additional file 1: Table S2.** Basic characteristics of the included studies.

**Additional file 2: Table S3.** The selection of the effect model.

**Additional file 3: Figure S7.** Evidence network map of CO.

**Additional file 4: Figure S8.** Direct comparison forest map of CO [MD(95%CI)].

**Additional file 5: Figure S9.** Network Meta-analysis heat map of CO [MD(95%CI)].

**Additional file 6: Figure S10** Probability ranking curves of the degree of improvement of CO [MD(95%CI)].

**Additional file 7: Figure S11.** Evidence network map of HDL-C.

**Additional file 8: Figure S12.** Direct comparison forest map of HDL-C [MD(95%CI)].

**Additional file 9: Figure S13.** Network Meta-analysis heat map of HDL-C [MD(95%CI)].

**Additional file 10: Figure S14.** Probability ranking curves of the degree of improvement of HDL-C [MD(95%CI)].

**Additional file 11: Figure S15.** Evidence network map of TG.

**Additional file 12: Figure S16.** Direct comparison forest map of TG [MD(95%CI)].

**Additional file 13: Figure S17.** Network Meta-analysis heat map of TG [MD(95%CI)].

**Additional file 14: Figure S18.** Probability ranking curves of the degree of TG reduction [MD(95%CI)].

**Additional file 15: Figure S19.** Evidence network map of LDL-C.

**Additional file 16: Figure S20.** Direct comparison forest map of LDL-C [MD(95%CI)].

**Additional file 17: Figure S21.** Network Meta-analysis heat map of LDL-C [MD(95%CI)].

**Additional file 18: Figure S22.** Probability ranking curves of the degree of LDL-C reduction [MD(95%CI)].

**Additional file 19: Figure S23.** Evidence network map of TC.

**Additional file 20: Figure S24.** Direct comparison forest map of TC [MD(95%CI)].

**Additional file 21: Figure S25.** Network Meta-analysis heat map of TC [MD(95%CI)].

**Additional file 22: Figure S26.** Probability ranking curves of the degree of TC reduction [MD(95%CI)].

**Additional file 23: Figure S27.** Evidence network map of total clinical effectiveness rate.

**Additional file 24: Figure S28.** Direct comparison forest map of total clinical effectiveness rate [MD(95%CI)].

**Additional file 25: Figure S29.** Network Meta-analysis heat map of total clinical effectiveness rate [MD(95%CI)].

**Additional file 26: Figure S30.** Probability ranking curves of the degree of improvement of total clinical effectiveness rate [MD (95%CI)].

**Additional file 27: Table S4.** Adverse reaction statistics table.

**Additional file 28: Table S5.** Table of subgroup analysis.

**Additional file 29: Figure S31.** Funnel plot of the risk of publication bias.

**Additional file 30.** Abbreviations.

**Additional file 31. ** Drug approval number.

## Data Availability

All data are available in the manuscript and they are showed in figures and tables.
