# Supplementary material for: Efficacy and safety of oral Chinese patent medicines in the treatment of coronary heart disease combined with hyperlipidemia: a systematic review and network meta-analysis of 78 trials
Source: Chin Med. 2023 Dec 13;18:162. doi: 10.1186/s13020-023-00866-x (PMC10717272; doi:10.1186/s13020-023-00866-x)
Supplement: Supplementary file 1 — Additional file 1: Table S2. Basic characteristics of the included studies. [file 13020_2023_866_MOESM1_ESM.docx]

**Table S2** Basic characteristics of the included studies

| Study | Average age (T/C) | Sample size (T/C) | Gender  (man/woman) | | Intervention | | Course of treatment  (days) | Outcome |
| --- | --- | --- | --- | --- | --- | --- | --- | --- |
|  |  |  | T | C | T | C |  |  |
| Li and Yan (2008) | 64.13 ± 2.76  /  63.88 ± 2.77 | 32/32 | 16/16 | 16/16 | CT+NXT | CT | 56 | ③④⑤⑥⑧ |
| Hao et al. (2016) | 60/53 | 95/95 | 62/33 | 58/37 | CT+NXT | CT | 30 | ③④⑤⑥⑦⑧ |
| Wu (2011) | 59/52 | 50/50 | 34/16 | 30/20 | CT+NXT | CT | 30 | ③④⑤⑥⑧ |
| Chen et al. (2016) | 57.0 ± 7.5/  54.0 ± 6.2 | 40/40 | 20/20 | 22/18 | CT+NXT | CT | 84 | ③④⑤⑥ |
| Bai (2012) | 68.3 ± 10.1/  68.5 ± 10.2 | 34/34 | 18/16 | 20/14 | CT+NXT | CT | 30 | ①②③④⑤⑥⑦ |
| Chen (2019) | - | 54/54 | - | - | CT+DSDW | CT | 90 | ①②③④⑤⑥⑦ |
| Deng (2015) | 61.58 ± 7.30/  61.16 ± 7.15 | 49/48 | 33/16 | 31/17 | CT+DSDW | CT | 84 | ③④⑤⑥⑦⑧ |
| Chen (2022) | 63.97 ± 5.48/  64.38 ± 5.79 | 43/43 | 25/18 | 26/17 | CT+DSDW | CT | 84 | ①  ②③④⑤⑥⑦ |
| Wang et al. (2015a) | 57.1 ± 8.2/  56.6 ± 7.1 | 97/97 | 59/38 | 56/41 | CT+DSDW | CT | 56 | ①②③④⑤⑥⑦ |
| Zhang et al. (2019a) | 65.8 ± 9.7/  67.5 ± 7.6 | 52/57 | 27/25 | 36/21 | CT+DSDW | CT | 90 | ①②③④⑤⑥ |
| Wang and Hu (2018) | 68.5 ± 4.1/  69.6 ± 4.5 | 49/49 | 27/22 | 24/25 | CT+DSDW | CT | 90 | ①②③④⑤⑥⑦ |
| Gao (2021) | 58.21 ± 4.77/  58.65 ± 4.21 | 56/56 | 31/25 | 30/26 | CT+DSDW | CT | 90 | ①②④⑤⑥ |
| Xie et al. (2018) | 57.08 ± 8.13/  57.25 ± 8.10 | 60/60 | 39/21 | 37/23 | CT+DSDW | CT | 60 | ①②⑦ |
| Liu (2014) | 65.4 ± 5.1/  64.8 ± 3.7 | 45/45 | 29/16 | 26/19 | CT+DSDW | CT | 90 | ①②⑤⑥⑦ |
| Li (2018a) | 57.23 ± 12.45  /  58.92 ± 13.45 | 60/60 | 37/23 | 36/24 | CT+DSDW | CT | 60 | ①④⑤⑥⑦ |
| Li (2017a) | 62.5 ± 5.1/  61.9 ± 3.6 | 50/50 | 30/20 | 28/22 | CT+DSDW | CT | 28 | ⑤⑥⑦ |
| Xu (2014a) | 62.1 ± 7.7/  63.5 ± 6.9 | 91/91 | 58/33 | 55/36 | CT+DSDW | CT | 56 | ①②③④⑤⑥⑦ |
| Zhao (2018) | 61.82 ± 4.90/  61.95 ± 4.92 | 46/46 | 27/19 | 26/20 | CT+DSDW | CT | 60 | ⑤⑥⑦ |
| Wu (2020b) | 65.42 ± 2.44/  66.42 ± 3.21 | 40/40 | 23/17 | 21/19 | CT+DSDW | CT | 60 | ③④⑤⑥⑦ |
| Shen (2020) | 61.3 ± 7.9/  61.1 ± 7.7 | 52/52 | 29/23 | 30/22 | CT+DSDW | CT | 56 | ①②④⑤⑥ |
| Li (2017b) | 59.2 ± 6.1/  60.4 ± 5.4 | 48/48 | 29/19 | 27/21 | CT+DSDW | CT | 84 | ①②③④⑤⑥⑦ |
| Guo (2016) | 57.4 ± 5.2/  56.1 ± 4.8 | 38/38 | 21/17 | 25/13 | CT+DSDW | CT | 84 | ④⑥⑦ |
| Xie et al. (2020) | 56.18 ± 4.24/  56.24 ± 4.21 | 53/53 | 32/21 | 34/19 | CT+DSDW | CT | 28 | ③④⑤⑥⑦ |
| Chen et al. (2017) | 68.3/  69.4 | 42/37 | 28/14 | 25/12 | CT+DSDW | CT | 84 | ⑤⑥⑦ |
| Rong (2014) | - | 40/40 | - | - | CT+DSDW | CT | 28 | ③④⑤⑥⑦ |
| Li (2019) | - | 88/78 | - | - | CT+DSDW | CT | 180 | ②③④⑤⑥⑦⑧ |
| Zhang (2013a) | 43.4 ± 10.4/  43.4 ± 10.4 | 40/40 | 21/19 | 22/18 | CT+DSDW | CT | 140 | ⑤⑥⑦ |
| Wang (2015b) | - | 50/50 | - | - | CT+NXT | CT | 56 | ③④⑤⑥⑦ |
| Gao et al. (2007) | 65.5 ± 9.2/  64.4 ± 9.8 | 45/45 | 30/15 | 26/19 | CT+NXT | CT | 28 | ①④⑤⑥⑧ |
| Zhang et al. (2013b) | 58.2 ± 7.5/  57.6 ± 7.9 | 40/40 | 21/19 | 23/17 | CT+NXT | CT | 84 | ③④⑤⑥⑦ |
| Gao et al. (2011) | 71.8/  72.4 | 64/62 | 42/22 | 39/23 | CT+NXT | CT | 56 | ③④⑤⑥⑦⑧ |
| Li (2016) | 69.4/  70.31 | 40/40 | 22/18 | 24/16 | CT+NXT | CT | 28 | ③④⑤⑥⑦⑧ |
| Lu(2014) | - | 44/44 | - | - | CT+NXT | CT | 60 | ③④⑤⑥⑦⑧ |
| Duan et al. (2015) | 67.8 ± 6.6/  68.2 ± 7.1 | 50/50 | 30/20 | 31/19 | CT+NXT | CT | 28 | ③④⑤⑥ |
| Zhang et al. (2014) | 67.9 ± 6.6/  67.6 ± 6.4 | 56/60 | 28/28 | 32/28 | CT+NXT | CT | 28 | ③④⑤⑥⑦⑧ |
| Wan et al. (2018) | 60.4 ± 11.2/  59.2 ± 10.6 | 62/58 | 38/24 | 36/22 | CT+NXT | CT | 28 | ①②④⑤⑥⑦ |
| Zhang (2017) | 55.2 ± 8.2/  52.1 ± 7.9 | 45/45 | 22/23 | 21/24 | CT+TXL | CT | 90 | ④⑤⑥⑦ |
| Sun (2018) | 60.78 ± 10.24/  61.23 ± 10.76 | 39/39 | 23/16 | 24/15 | CT+TXL | CT | 90 | ①④⑤⑦ |
| Wu (2020a) | 55.42 ± 5.72/  55.36 ± 5.69 | 40/40 | 23/17 | 24/16 | CT+DSDW | CT | 30 | ③④⑤⑥ |
| Zhou (2017) | - | 35/35 | - | - | CT+TXL | CT | 90 | ③④⑤⑥⑦ |
| Peng (2021) | 65.24 ± 5.40  /  64.81 ± 5.63 | 50/50 | 26/24 | 25/25 | CT+SXBXW | CT | 90 | ①④⑤⑥⑦ |
| Zhang (2019b) | 64.59 ± 8.62/  63.91 ± 8.82 | 49/49 | 30/19 | 33/16 | CT+SXBXW | CT | 56 | ④⑤⑥⑦ |
| Cao (2020) | 60.18 ± 5.77/  60.07 ± 5.31 | 50/50 | 26/24 | 29/21 | CT+SXBXW | CT | 84 | ④⑥ |
| Song et al. (2017) | 59.2 ± 6.1/  60.4 ± 5.4 | 96/96 | 53/43 | 47/49 | CT+SXBXW | CT | 84 | ①②③④⑤⑥⑦ |
| Hu (2021) | 61.23 ± 3.01/  62.33 ± 3.58 | 43/43 | 26/17 | 24/19 | CT+SXBXW | CT | 90 | ④⑥⑦⑧ |
| Hu et al. (2012) | 61.4 ± 7.8/  61.4 ± 7.6 | 59/50 | 34/25 | 30/20 | CT+SXBXW | CT | 28 | ③④⑤⑥⑦ |
| Cai et al. (2015) | 63 ± 5/  61 ± 7 | 48/48 | 26/22 | 29/19 | CT+XMK | CT | 84 | ③④⑤⑥⑦⑧ |
| Yang (2014) | 51.3 ± 10.2/  52.5 ± 10.5 | 35/35 | 19/16 | 20/15 | CT+XMK | CT | 28 | ③④⑤⑥⑧ |
| Li (2018b) | 65.35 ± 6.04/  65.22 ± 6.11 | 40/40 | 21/19 | 20/20 | CT+XMK | CT | 28 | ③④⑤⑥⑦ |
| Zhang and Wei (2006) | 64.5 ± 10.2/  62.4 ± 10.9 | 30/30 | 19/11 | 18/12 | CT+TXL | CT | 28 | ③④⑤⑥⑦⑧ |
| Jiang (2016) | 63.2 ± 6.9/  63.1 ± 5.7 | 81/81 | 43/38 | 46/35 | CT+TXL | CT | 56 | ③④⑤⑥⑦ |
| Zhou (2021) | 59.61 ± 3.79/  59.62 ± 3.76 | 42/42 | 25/17 | 27/15 | CT+TXL | CT | 30 | ③④⑤⑥⑦ |
| Kou (2022) | 63.7 ± 6.9/  60.7 ± 5.6 | 50/50 | 27/23 | 28/22 | CT+TXL | CT | 30 | ①②③④⑤⑥⑦ |
| Li (2004) | 67/66 | 36/24 | 27/9 | 16/8 | CT+TXL | CT | 28 | ③④⑥⑦⑧ |
| He (2008a) | 56.5 ± 7.2/  58.3 ± 7.4 | 42/40 | 32/10 | 30/10 | CT+TXL | CT | 56 | ①②③④⑥ |
| Li and Zhu (2016) | 60.31 ± 6.56/  60.26 ± 6.44 | 31/31 | 19/12 | 20/11 | CT+TXL | CT | 28 | ④⑥⑦ |
| Wang (2016) | - | 50/50 | - | - | CT+TXL | CT | 28 | ⑦ |
| Zhang and Zhang (2005) | 60.2 ± 7.6/  60.5 ± 6.8 | 60/60 | 42/18 | 34/26 | CT+TXL | CT | 28 | ③④⑤⑥⑦⑧ |
| Cai (2006) | 61.4 ± 7.9/  61.4 ± 7.4 | 56/52 | 32/24 | 31/21 | CT+TXL | CT | 28 | ③④⑥⑦ |
| Chen (2004) | 61 ± 7/  60 ± 8 | 30/30 | 23/7 | 20/10 | CT+TXL | CT | 56 | ③④⑤⑥⑦⑧ |
| Ni (2014) | 65 ± 2.3/  64 ± 1.8 | 65/65 | 38/27 | 40/25 | CT+TXL | CT | 28 | ③④⑥⑦ |
| Jin and Xie (2019) | 45.8 ± 8.5/  46.7 ± 7.9 | 64/64 | 40/24 | 41/23 | CT+DSDW | CT | 84 | ④⑤⑥⑦ |
| Qu et al. (2020) | 50.08 ± 4.19/  51.08 ± 4.27 | 39/39 | 22/17 | 25/14 | CT+XZK | CT | 84 | ③⑤⑥⑦ |
| Qu (2015) | 64.8 ± 3.1/  65.3 ± 2.7 | 100/100 | 59/41 | 58/42 | CT+XZK | CT | 56 | ③④⑤⑥⑦⑧ |
| Zhao et al. (2008) | - | 30/30 | 17/13 | 18/12 | CT+XZK | CT | 63 | ③④⑤⑥⑧ |
| Huang et al. (2009) | 65.78 ± 4.62/  65.97 ± 4.89 | 43/42 | 22/21 | 22/20 | CT+XZK | CT | 84 | ③④⑤⑥⑧ |
| Qu et al. (2017) | 55.2 ± 6.5/  56.6 ± 5.2 | 80/80 | 52/28 | 47/33 | CT+YDXNT | CT | 84 | ③④⑤⑥⑦ |
| Pang et al. (2018) | 66.3 ± 3.6/  65.7 ± 3.2 | 41/41 | 26/15 | 27/14 | CT+YDXNT | CT | 28 | ③④⑤⑥ |
| Dong et al. (2012) | - | 38/38 | - | - | CT+YDXNT | CT | 56 | ③④⑤⑥⑦ |
| He et al. (2008b) | 66.9 ± 7.5/  66.7 ± 8.1 | 35/33 | 22/13 | 21/12 | CT+YDXNT | CT | 28 | ③④⑤⑥⑦⑧ |
| Chen (2013) | 63.91 ± 7.95/  61.49 ± 9.42 | 70/35 | 38/32 | 21/14 | CT+ZBT | CT | 84 | ③④⑤⑥⑦⑧ |
| Liu et al. (2019) | 73.9 ± 8.2/  72.7 ± 8.5 | 45/45 | 27/18 | 29/16 | CT+ZBT | CT | 365 | ③④⑤⑥⑦ |
| Shi (2008) | - | 35/35 | 19/16 | 20/15 | CT+ZBT | CT | 49 | ③④⑤⑥⑦⑧ |
| Dong (2017) | 62.5 ± 3.8/  63.1 ± 3.9 | 43/43 | 29/14 | 30/13 | CT+DSDW | CT | 56 | ①②④⑤⑥⑦ |
| Xu (2014b) | - | 99/98 | - | - | CT+DSDW | CT | 56 | ①②④⑤⑥⑦ |
| Hu (2005) | 61.4 ± 7.9/  61.4 ± 7.4 | 56/52 | 32/24 | 31/21 | CT+TXL | CT | 28 | ③④⑥⑦ |
| Wang (2021) | 64.5 ± 6.3/  65.8 ± 5.7 | 34/34 | 19/15 | 20/14 | CT+DSDW | CT | 56 | ④⑤⑥⑦ |
| Li et al. (2009) | 66.68 ± 4.23/  66.79 ± 4.48 | 60/60 | 40/20 | 38/22 | CT+XZK | CT | 56 | ④⑤⑥⑧ |

Note: T: treatment group; C: control group; ①: CI; ②: CO; ③: HDL-C; ④: TG; ⑤: LDL-C; ⑥: TC; ⑦: total clinical effectiveness rate;⑧: incidence of adverse reactions.
