# Supplementary material for: Efficacy and safety of oral Chinese patent medicines in the treatment of coronary heart disease combined with hyperlipidemia: a systematic review and network meta-analysis of 78 trials
Source: Chin Med. 2023 Dec 13;18:162. doi: 10.1186/s13020-023-00866-x (PMC10717272; doi:10.1186/s13020-023-00866-x)
Supplement: Supplementary file 2 — Additional file 2: Table S3. The selection of the effect model. [file 13020_2023_866_MOESM2_ESM.docx]

**Table S3** The selection of the effect model

| outcome indicator | DIC | | The selection of the effect model |
| --- | --- | --- | --- |
|  | fixed effect model | random effect model |  |
| CI | 1601.52 | 71.77 | random effect model |
| CO | 1315.04 | 75.76 | random effect model |
| HDL-C | 3829.06 | 228.86 | random effect model |
| TG | 3782.34 | 278.27 | random effect model |
| LDL-C | 2910.86 | 266.13 | random effect model |
| TC | 3491.35 | 300.54 | random effect model |
| Total clinical effectiveness rate | 174.49 | 175.54 | fixed effect model |
