# Supplementary figures and images for: Efficacy and safety of oral Chinese patent medicines in the treatment of coronary heart disease combined with hyperlipidemia: a systematic review and network meta-analysis of 78 trials
Source: Chin Med. 2023 Dec 13;18:162. doi: 10.1186/s13020-023-00866-x (PMC10717272; doi:10.1186/s13020-023-00866-x)

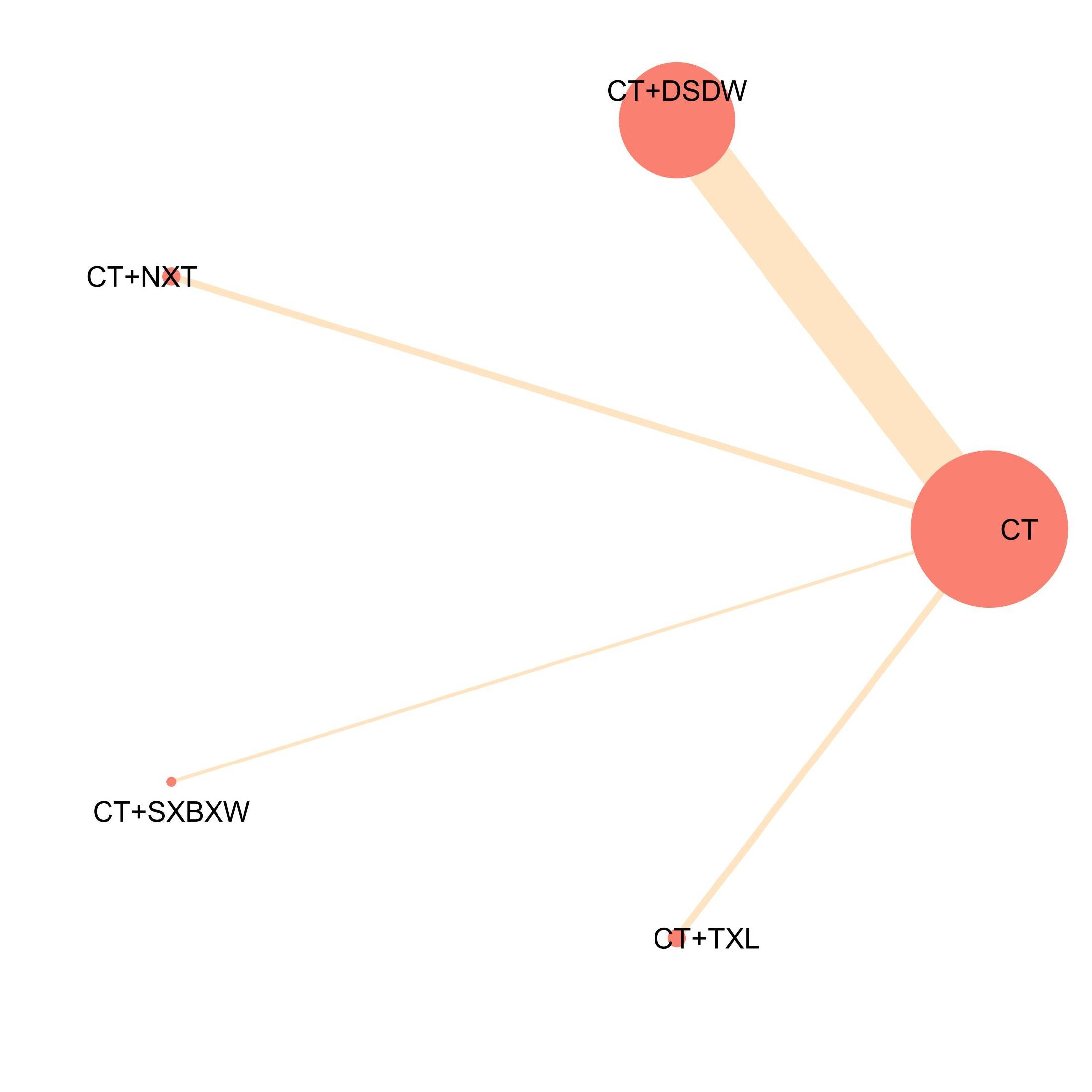

Supplement: Supplementary file 3 — Additional file 3: Figure S7. Evidence network map of CO. [file 13020_2023_866_MOESM3_ESM.png]

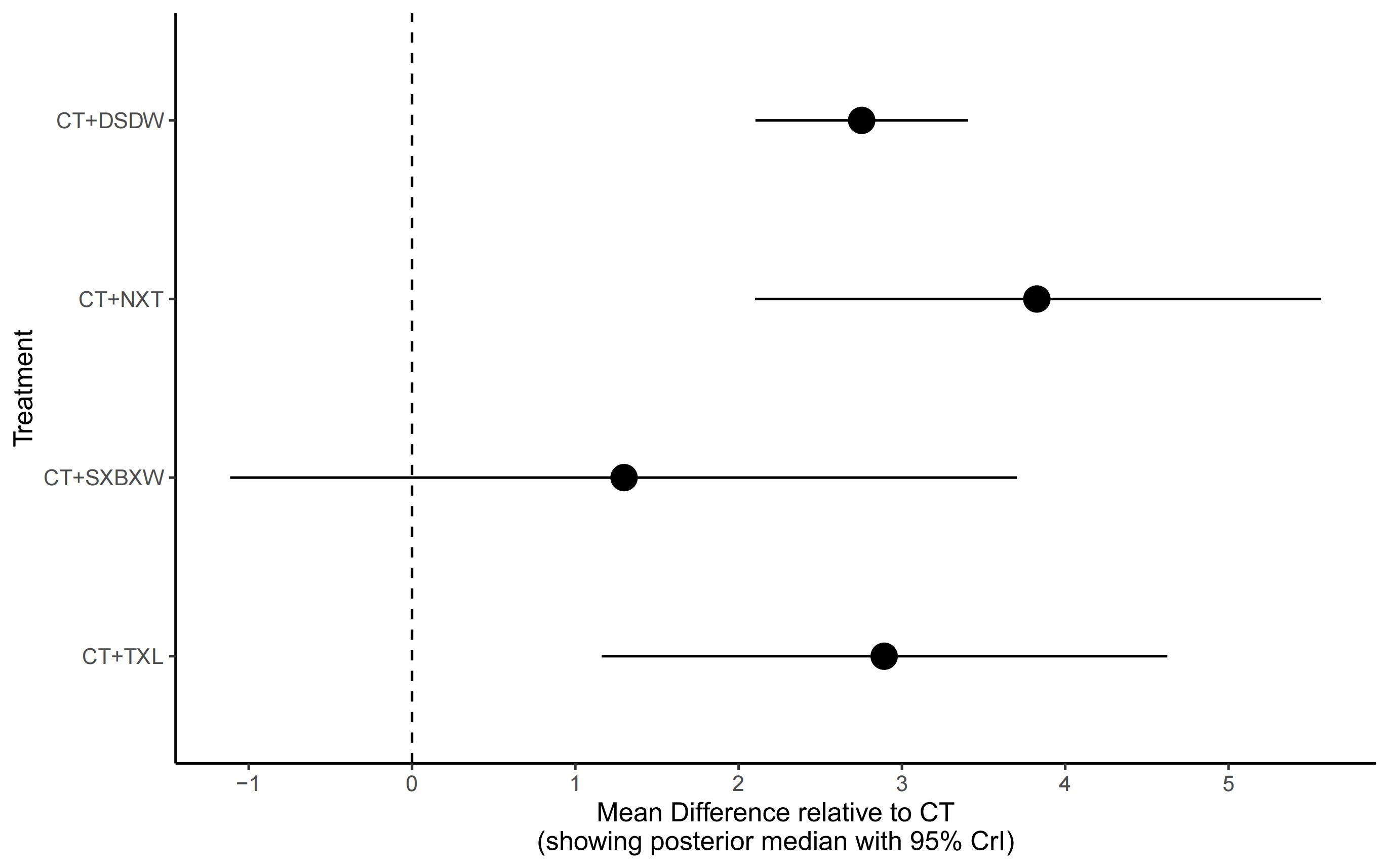

Supplement: Supplementary file 4 — Additional file 4: Figure S8. Direct comparison forest map of CO [MD(95%CI)]. [file 13020_2023_866_MOESM4_ESM.png]

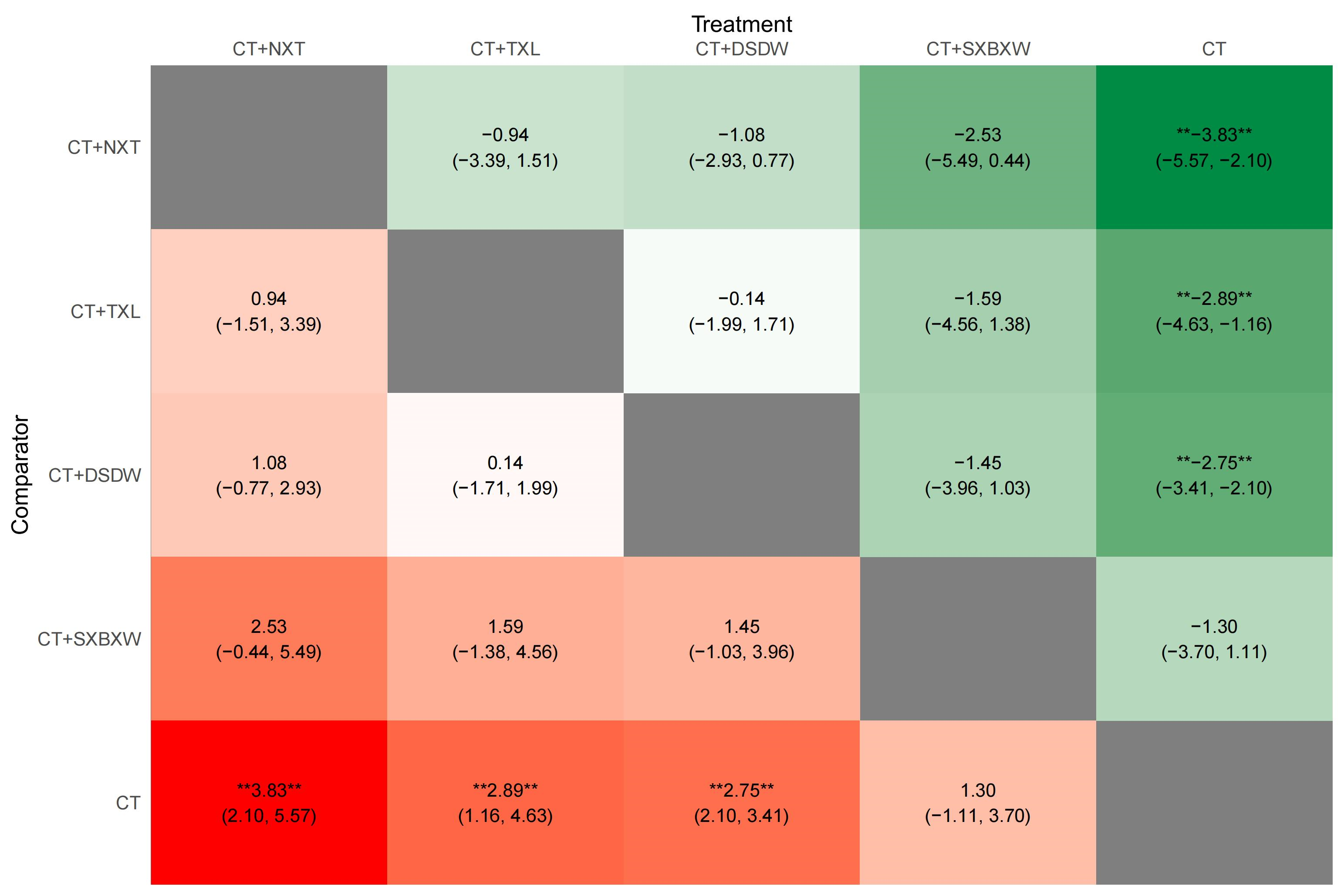

Supplement: Supplementary file 5 — Additional file 5: Figure S9. Network Meta-analysis heat map of CO [MD(95%CI)]. [file 13020_2023_866_MOESM5_ESM.png]

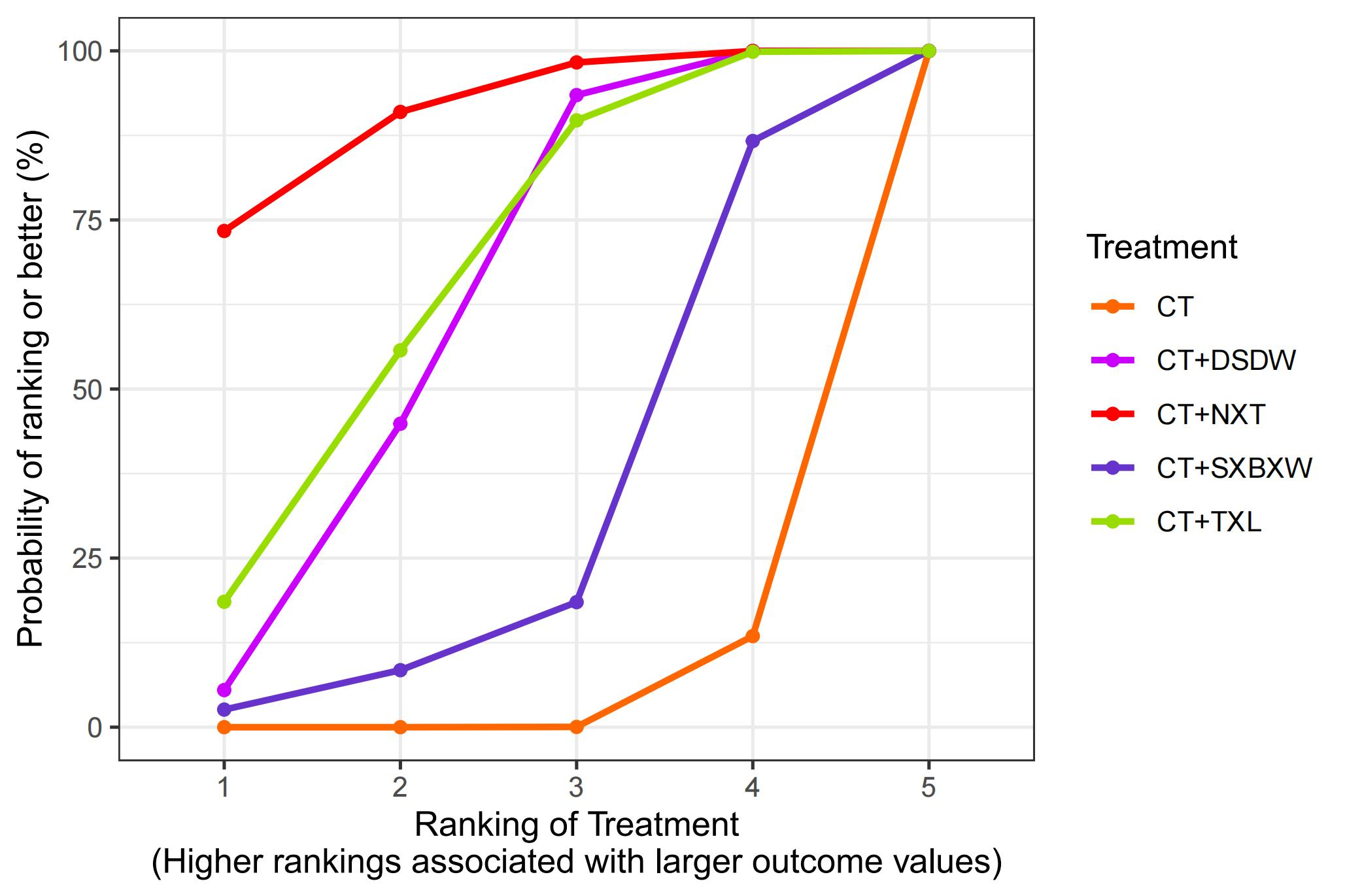

Supplement: Supplementary file 6 — Additional file 6: Figure S10 Probability ranking curves of the degree of improvement of CO [MD(95%CI)]. [file 13020_2023_866_MOESM6_ESM.png]

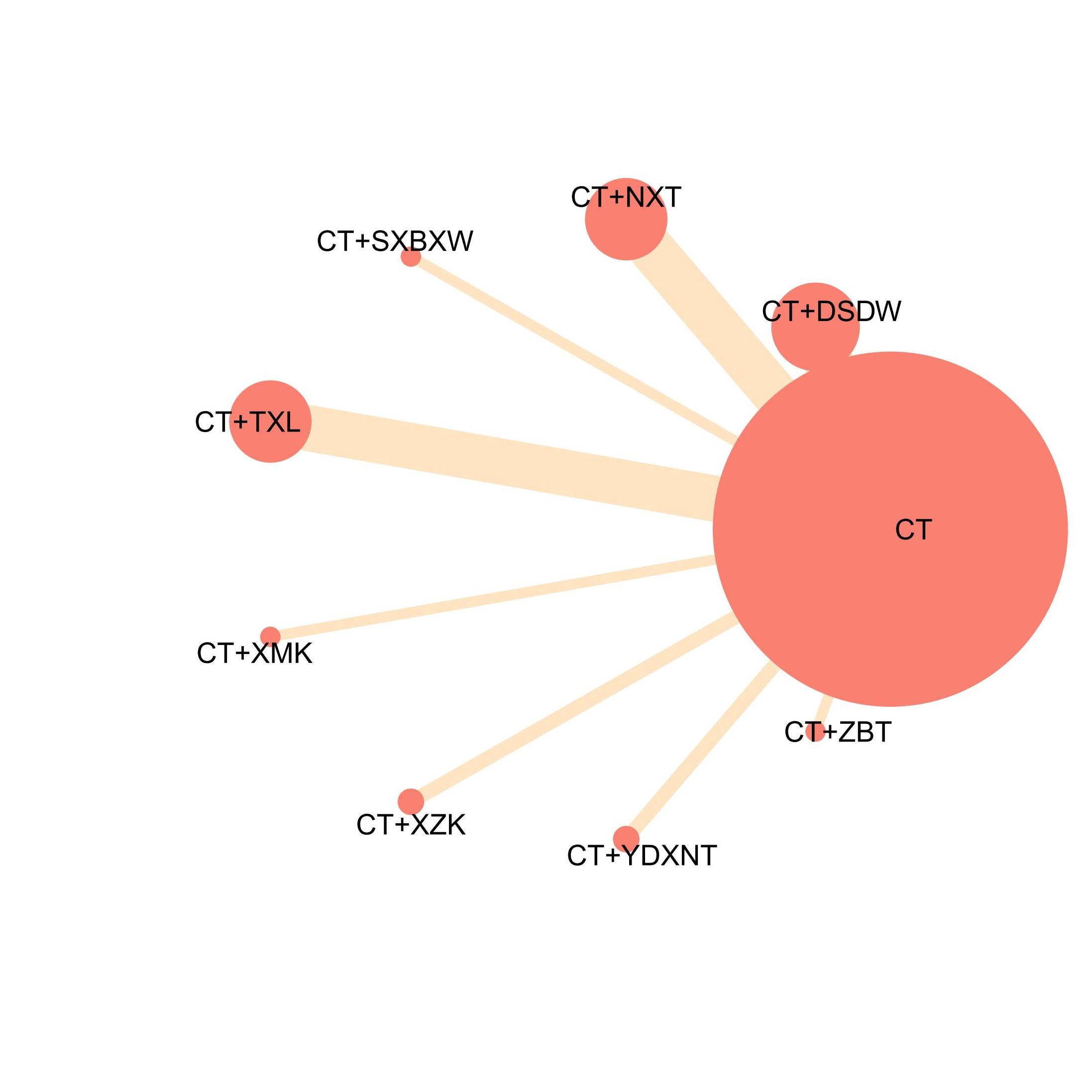

Supplement: Supplementary file 7 — Additional file 7: Figure S11. Evidence network map of HDL-C. [file 13020_2023_866_MOESM7_ESM.png]

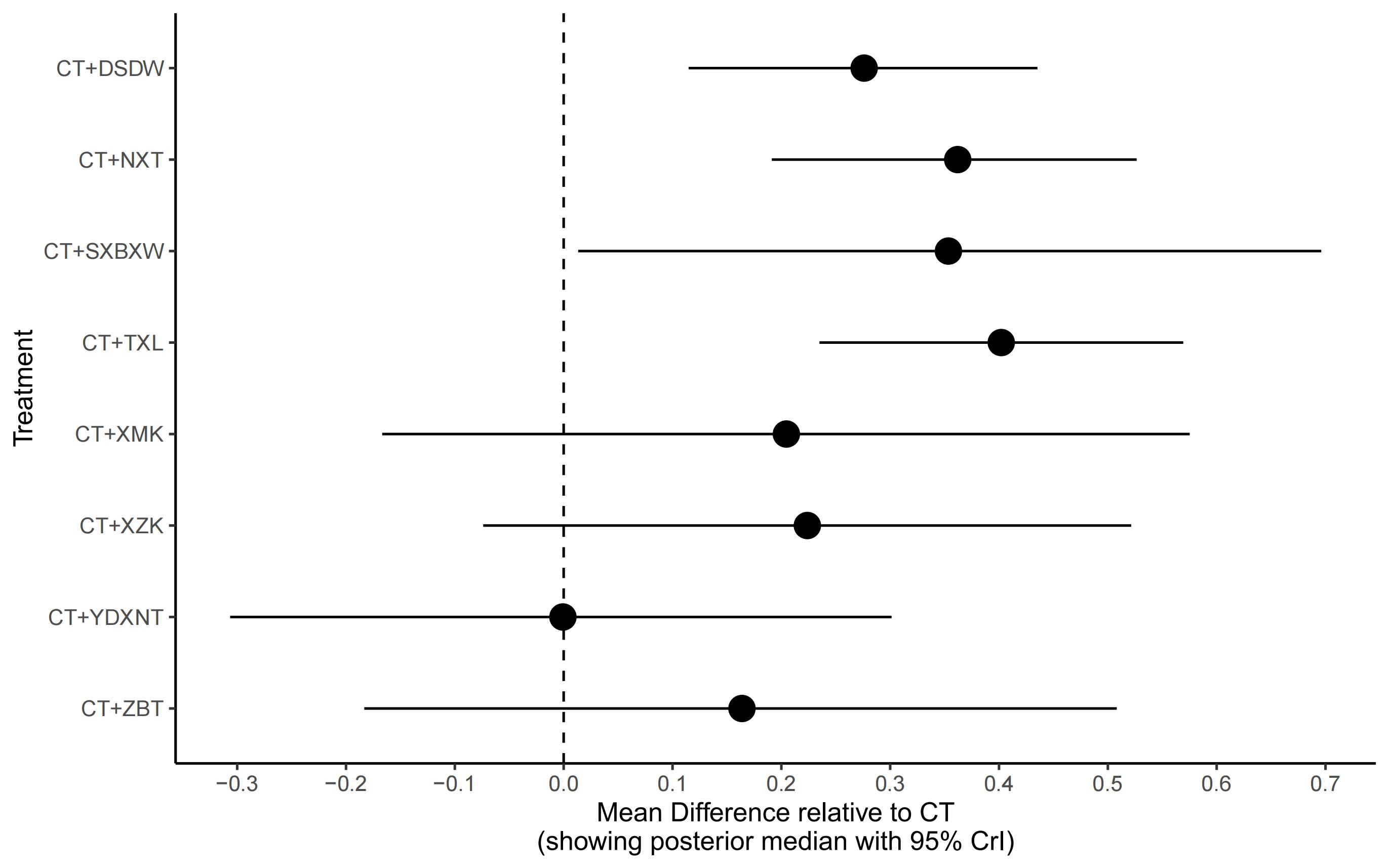

Supplement: Supplementary file 8 — Additional file 8: Figure S12. Direct comparison forest map of HDL-C [MD(95%CI)]. [file 13020_2023_866_MOESM8_ESM.png]

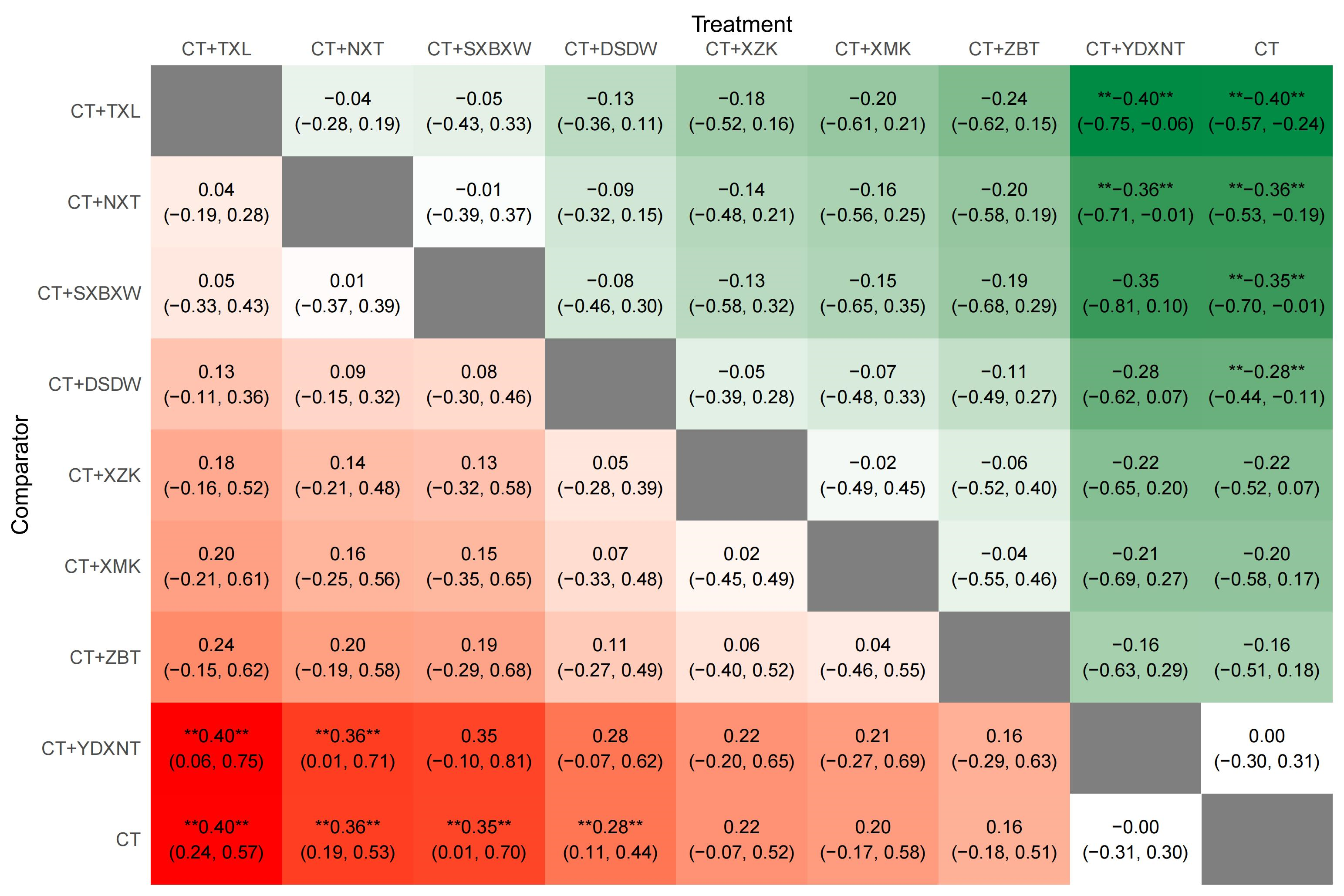

Supplement: Supplementary file 9 — Additional file 9: Figure S13. Network Meta-analysis heat map of HDL-C [MD(95%CI)]. [file 13020_2023_866_MOESM9_ESM.png]

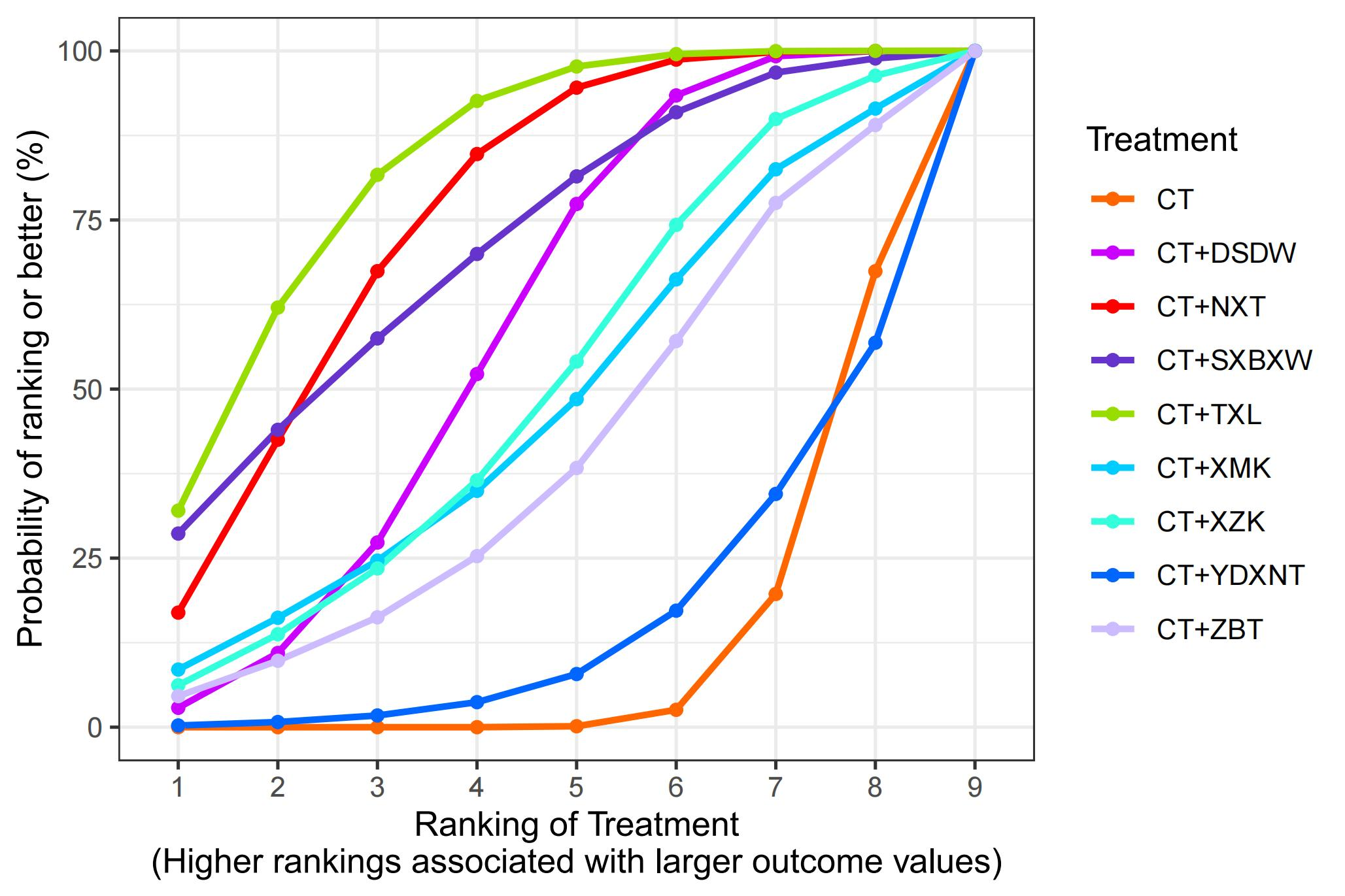

Supplement: Supplementary file 10 — Additional file 10: Figure S14. Probability ranking curves of the degree of improvement of HDL-C [MD(95%CI)]. [file 13020_2023_866_MOESM10_ESM.png]

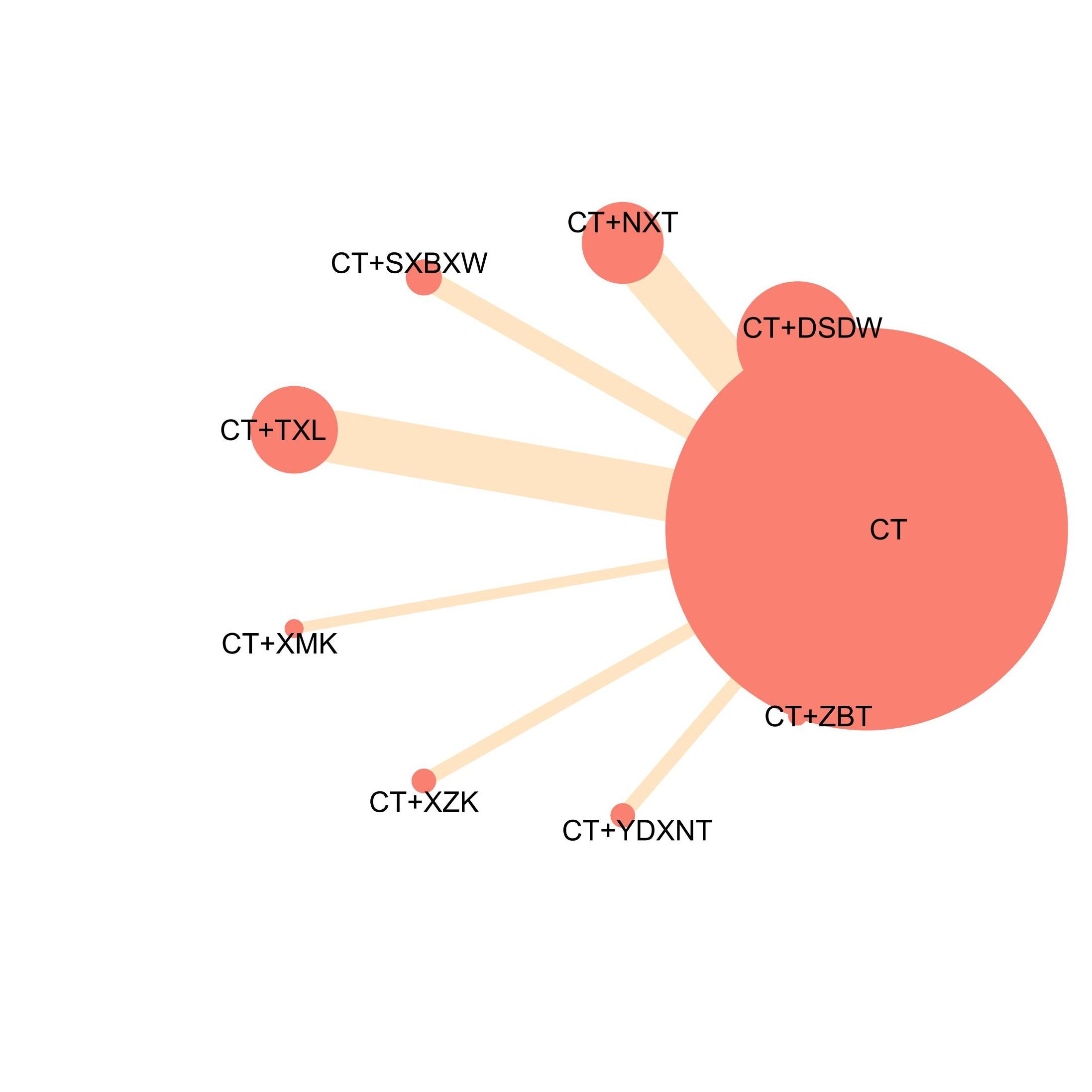

Supplement: Supplementary file 11 — Additional file 11: Figure S15. Evidence network map of TG. [file 13020_2023_866_MOESM11_ESM.png]

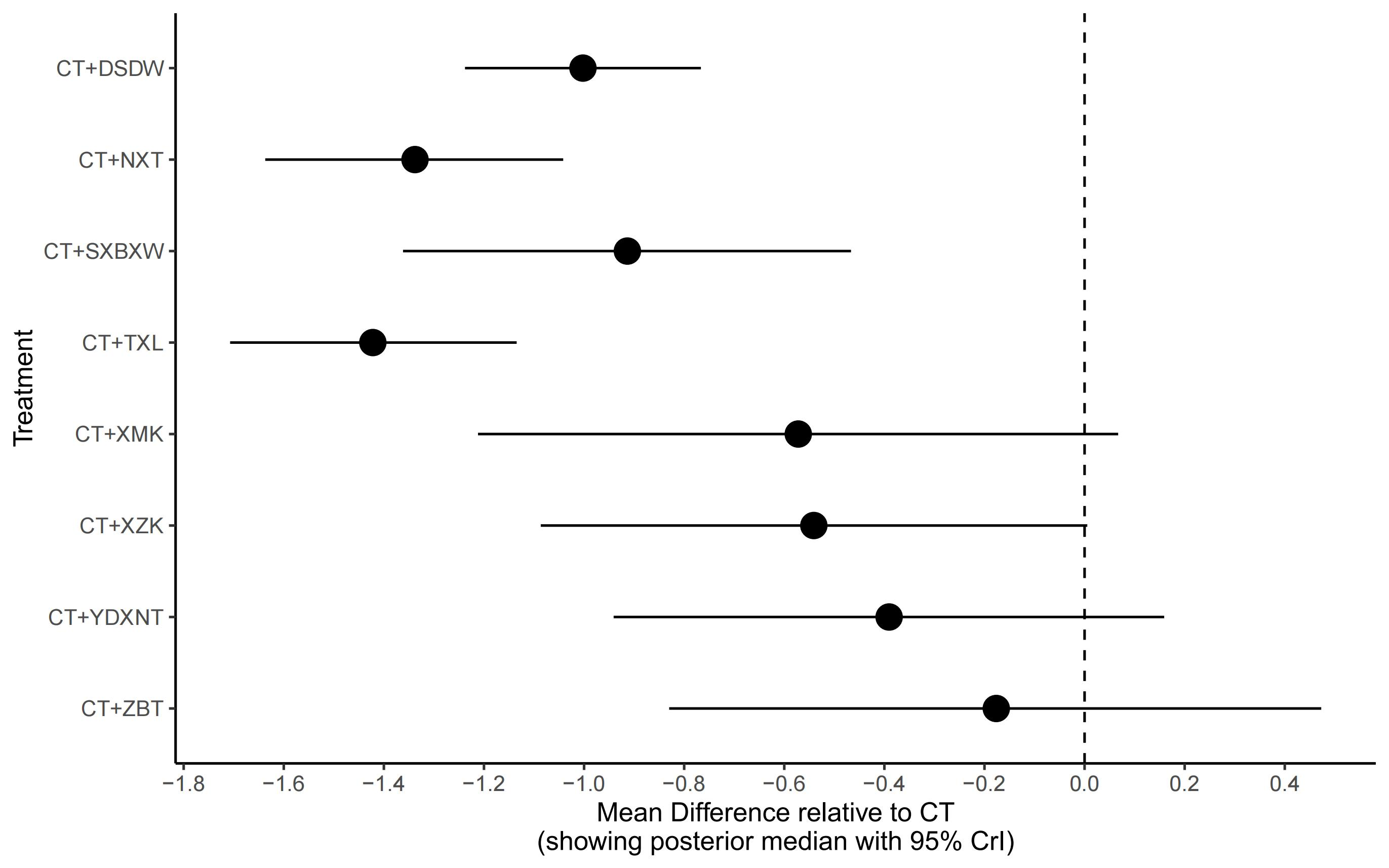

Supplement: Supplementary file 12 — Additional file 12: Figure S16. Direct comparison forest map of TG [MD(95%CI)]. [file 13020_2023_866_MOESM12_ESM.png]

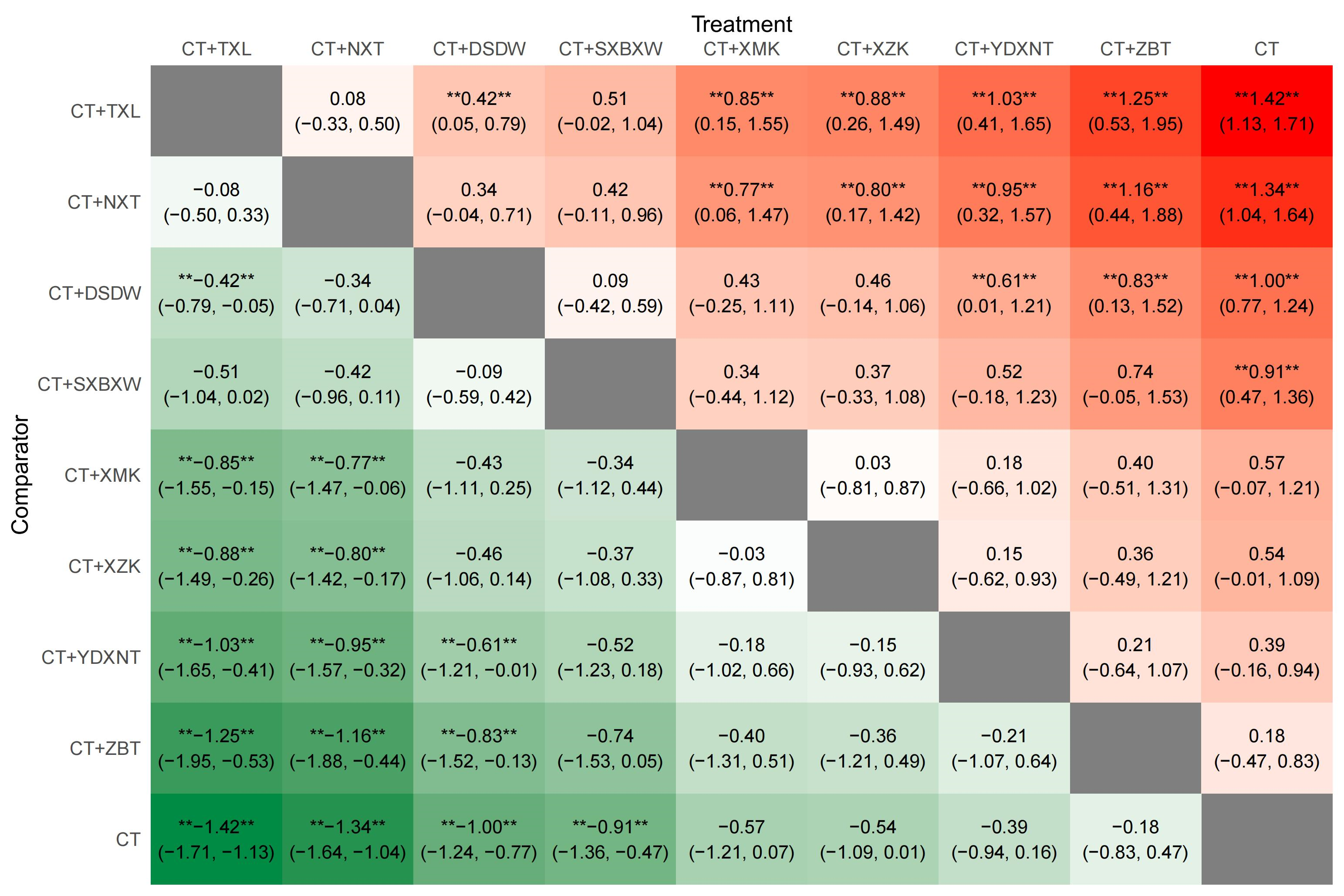

Supplement: Supplementary file 13 — Additional file 13: Figure S17. Network Meta-analysis heat map of TG [MD(95%CI)]. [file 13020_2023_866_MOESM13_ESM.png]

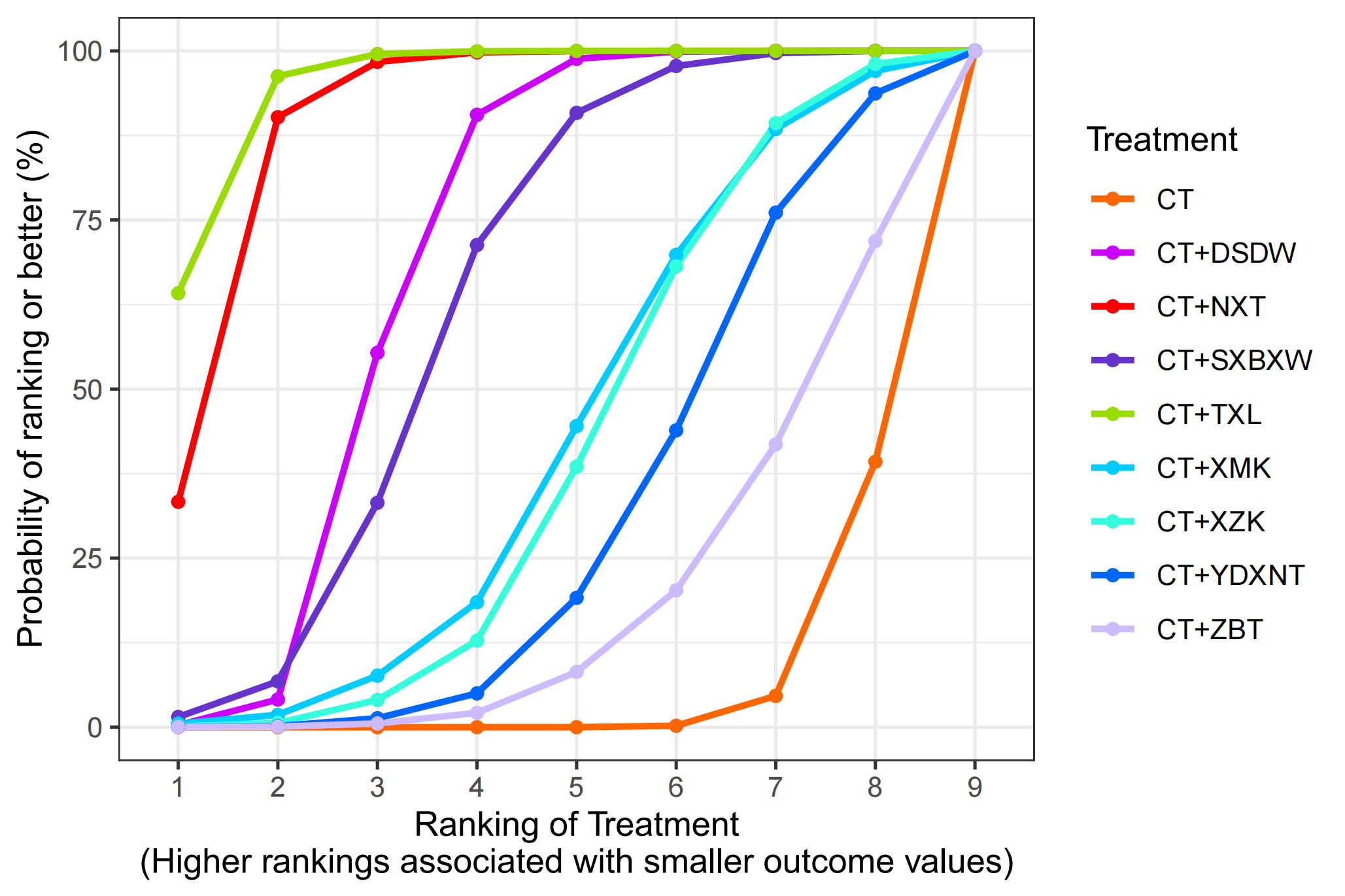

Supplement: Supplementary file 14 — Additional file 14: Figure S18. Probability ranking curves of the degree of TG reduction [MD(95%CI)]. [file 13020_2023_866_MOESM14_ESM.png]

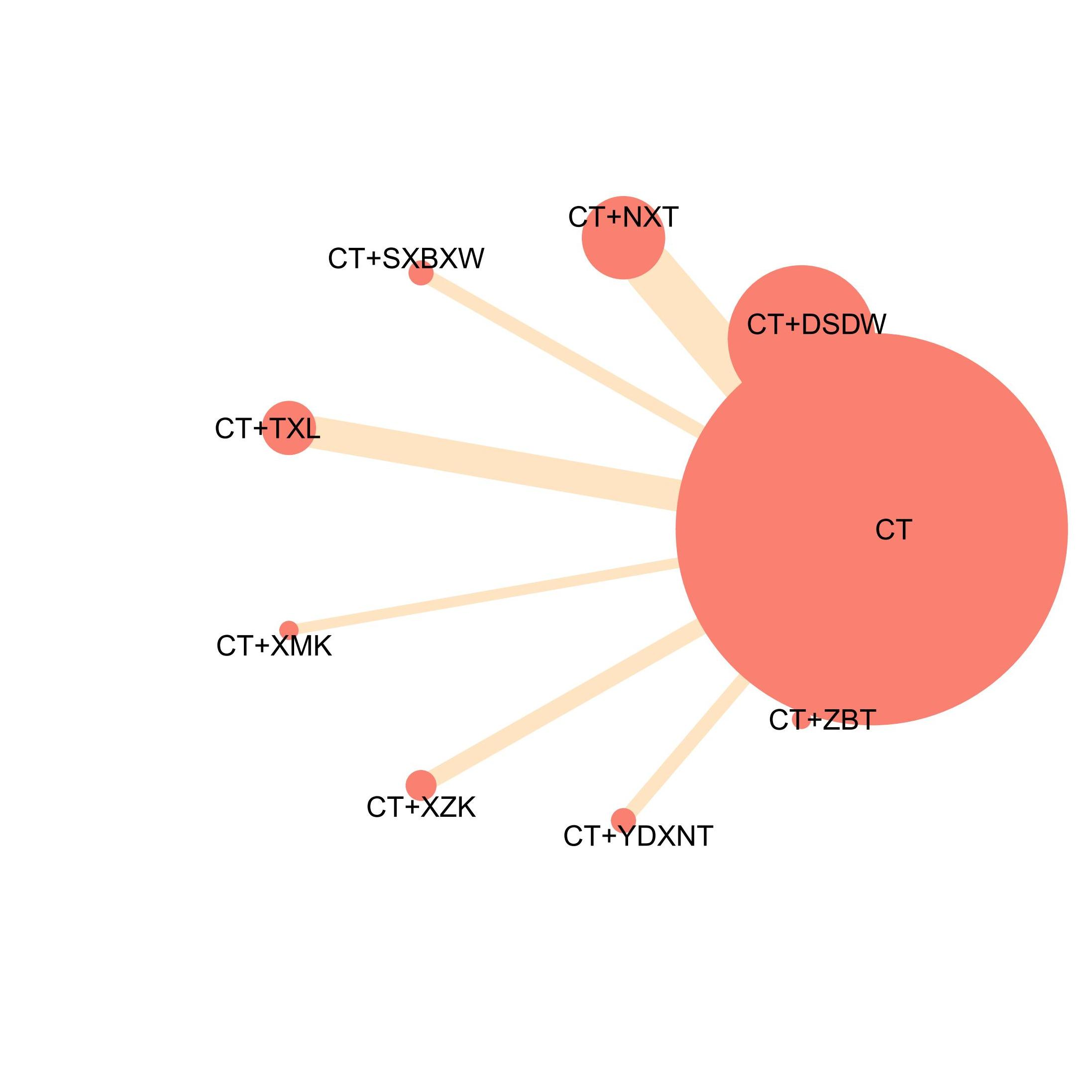

Supplement: Supplementary file 15 — Additional file 15: Figure S19. Evidence network map of LDL-C. [file 13020_2023_866_MOESM15_ESM.png]

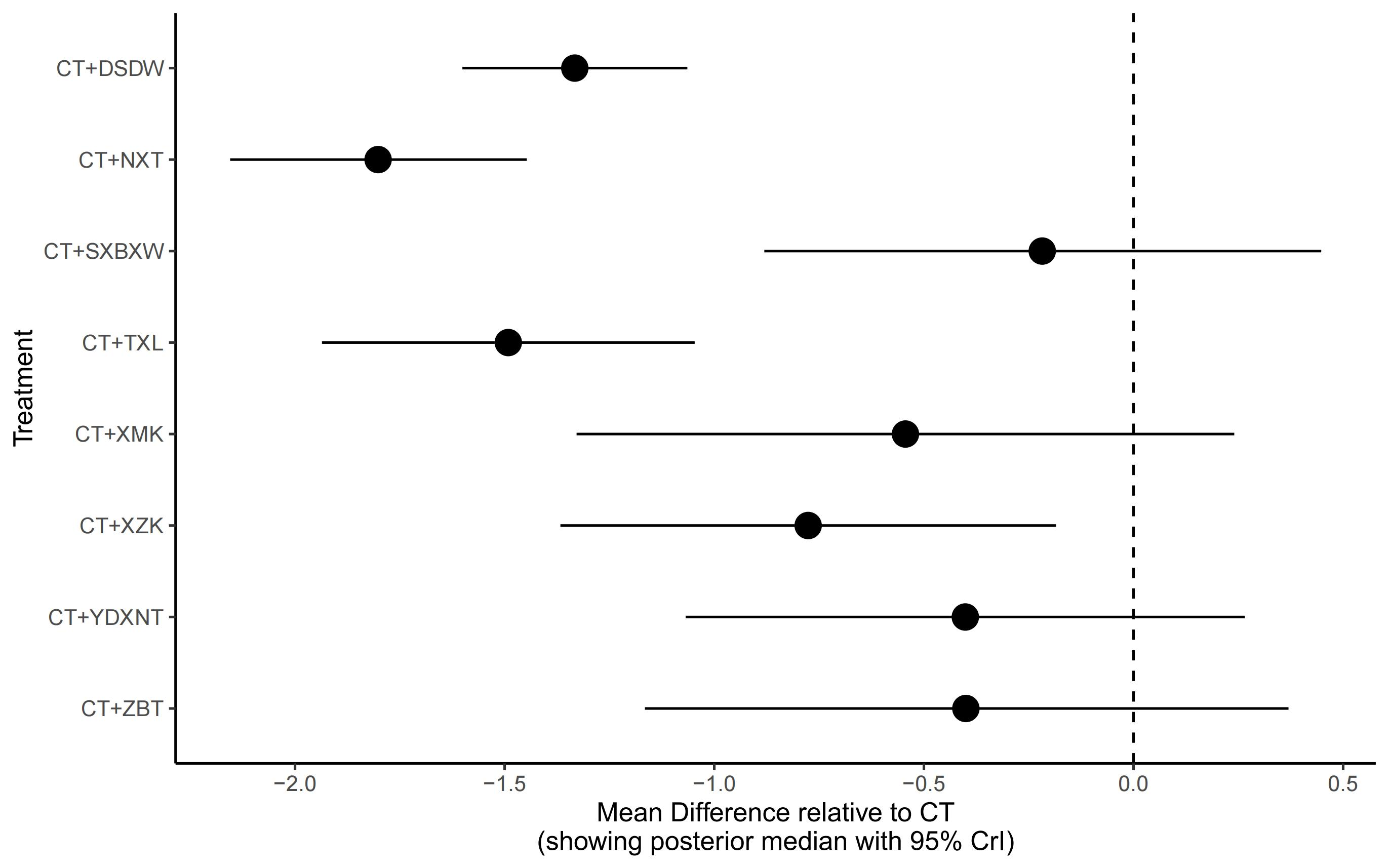

Supplement: Supplementary file 16 — Additional file 16: Figure S20. Direct comparison forest map of LDL-C [MD(95%CI)]. [file 13020_2023_866_MOESM16_ESM.png]

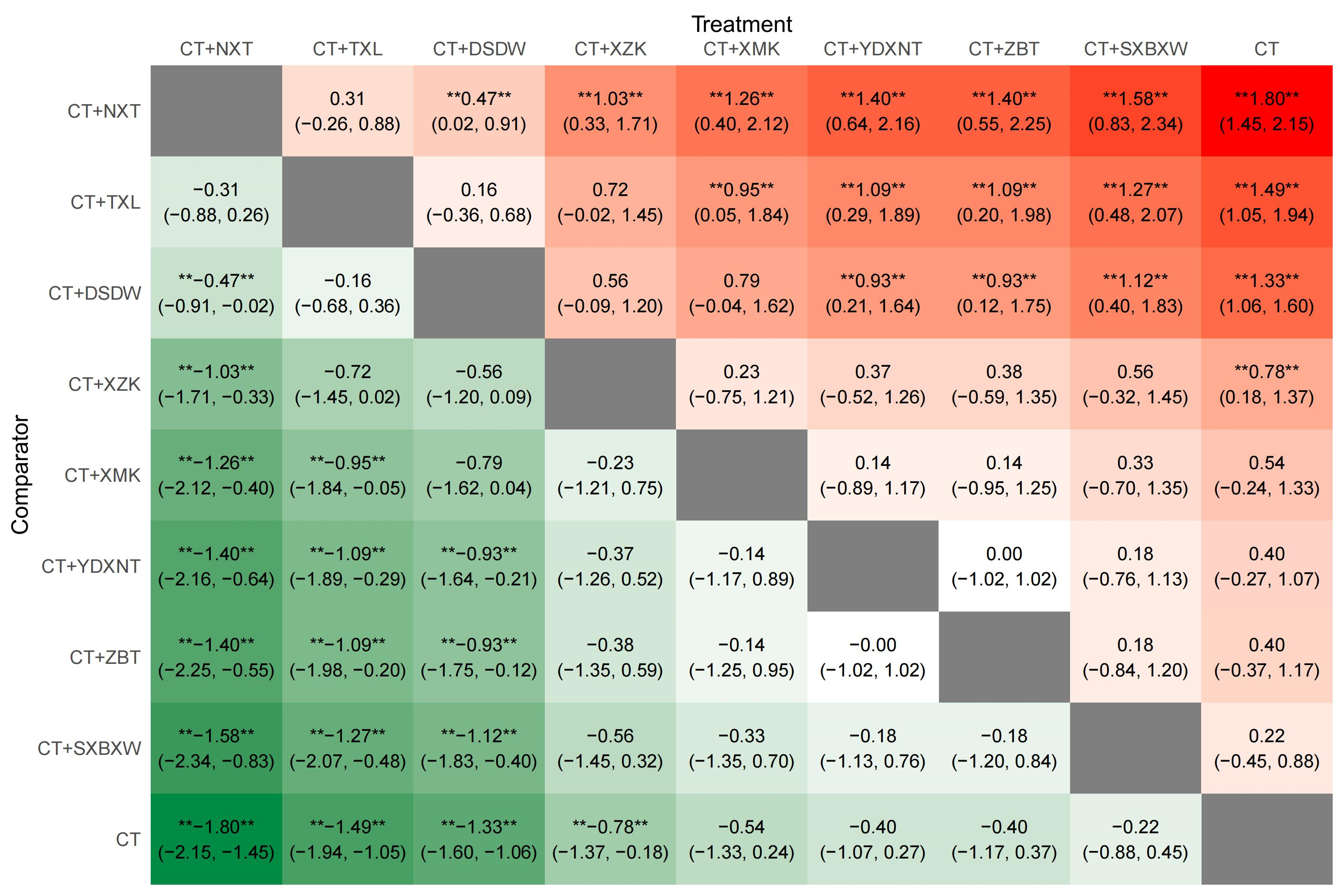

Supplement: Supplementary file 17 — Additional file 17: Figure S21. Network Meta-analysis heat map of LDL-C [MD(95%CI)]. [file 13020_2023_866_MOESM17_ESM.png]

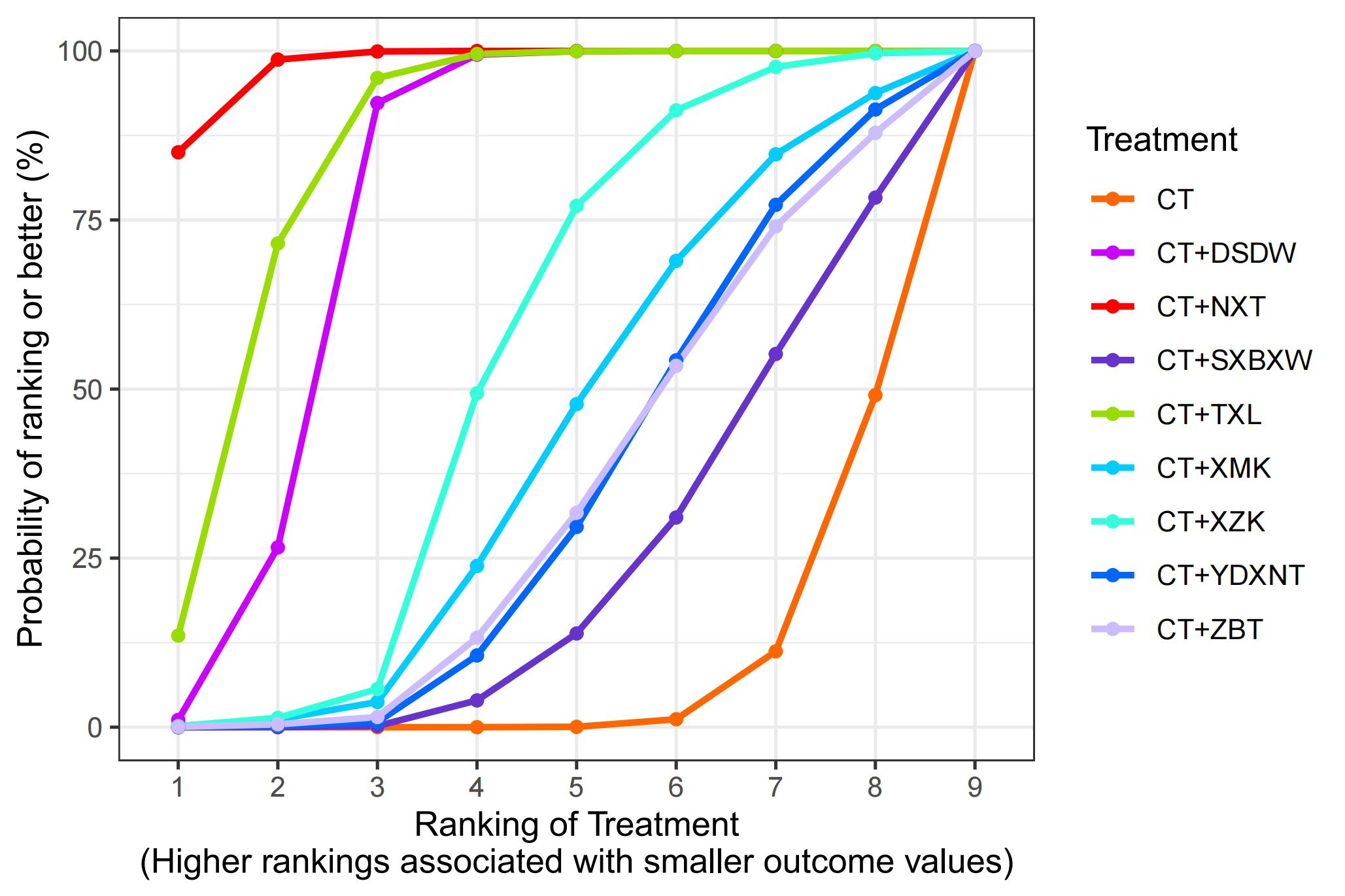

Supplement: Supplementary file 18 — Additional file 18: Figure S22. Probability ranking curves of the degree of LDL-C reduction [MD(95%CI)]. [file 13020_2023_866_MOESM18_ESM.png]

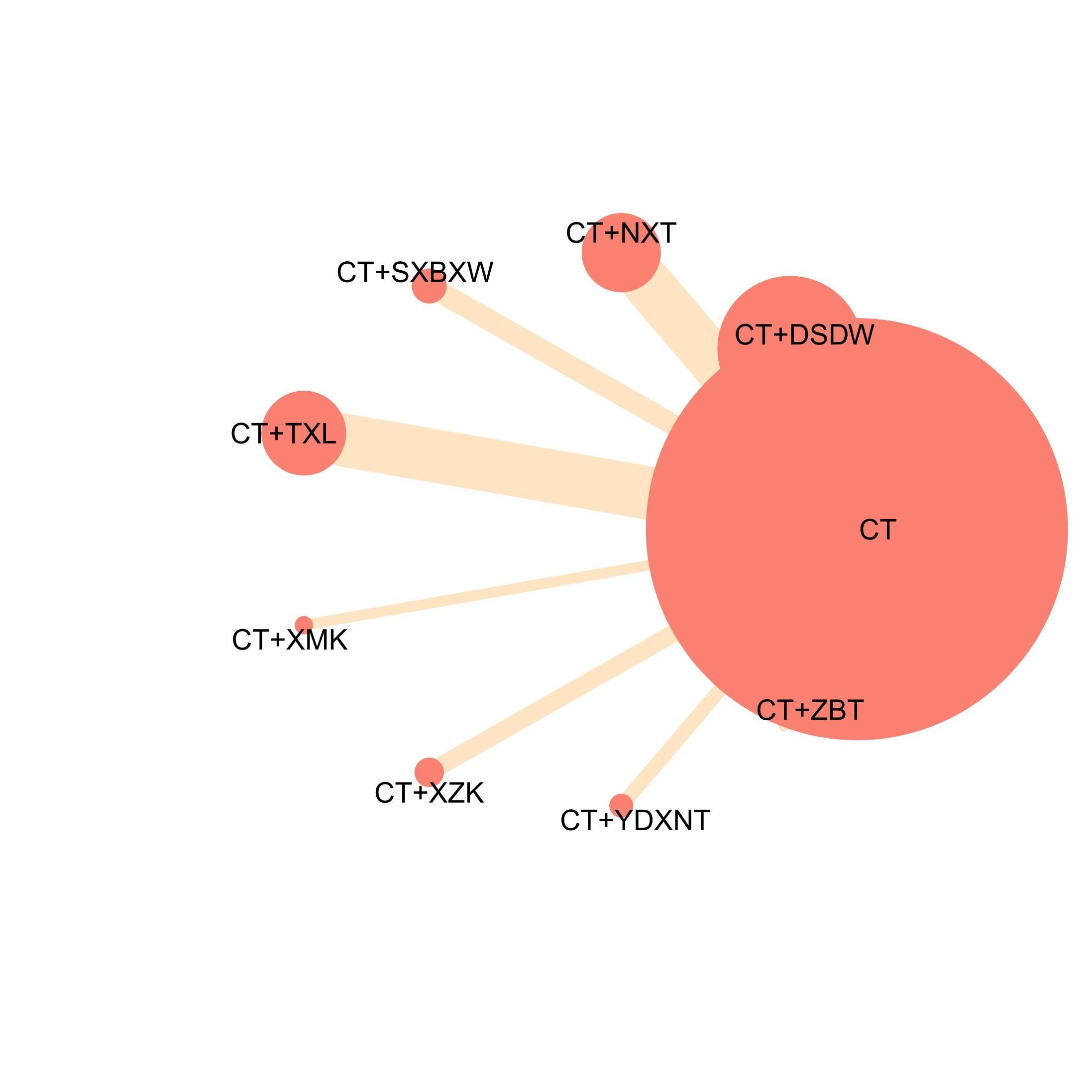

Supplement: Supplementary file 19 — Additional file 19: Figure S23. Evidence network map of TC. [file 13020_2023_866_MOESM19_ESM.png]

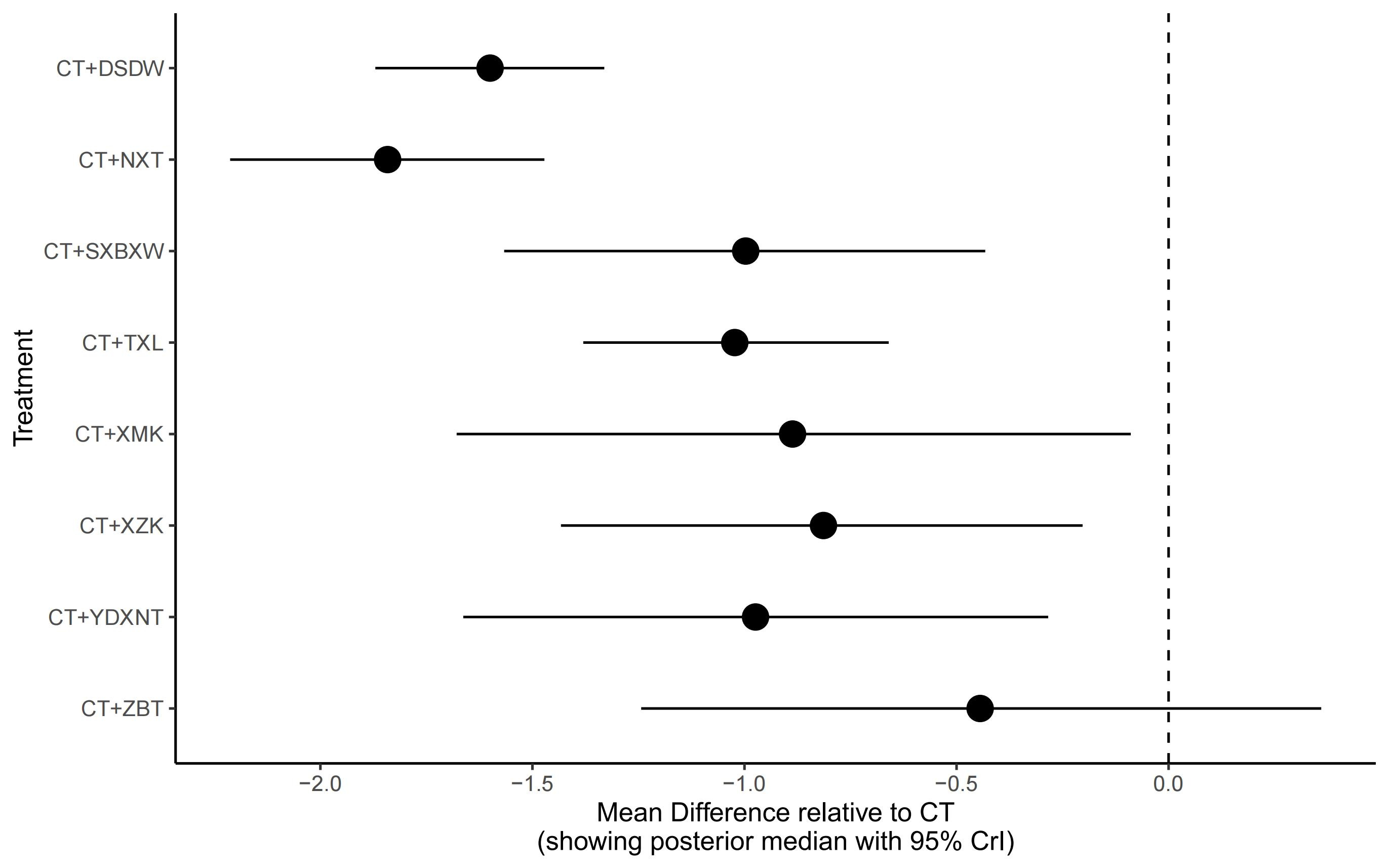

Supplement: Supplementary file 20 — Additional file 20: Figure S24. Direct comparison forest map of TC [MD(95%CI)]. [file 13020_2023_866_MOESM20_ESM.png]

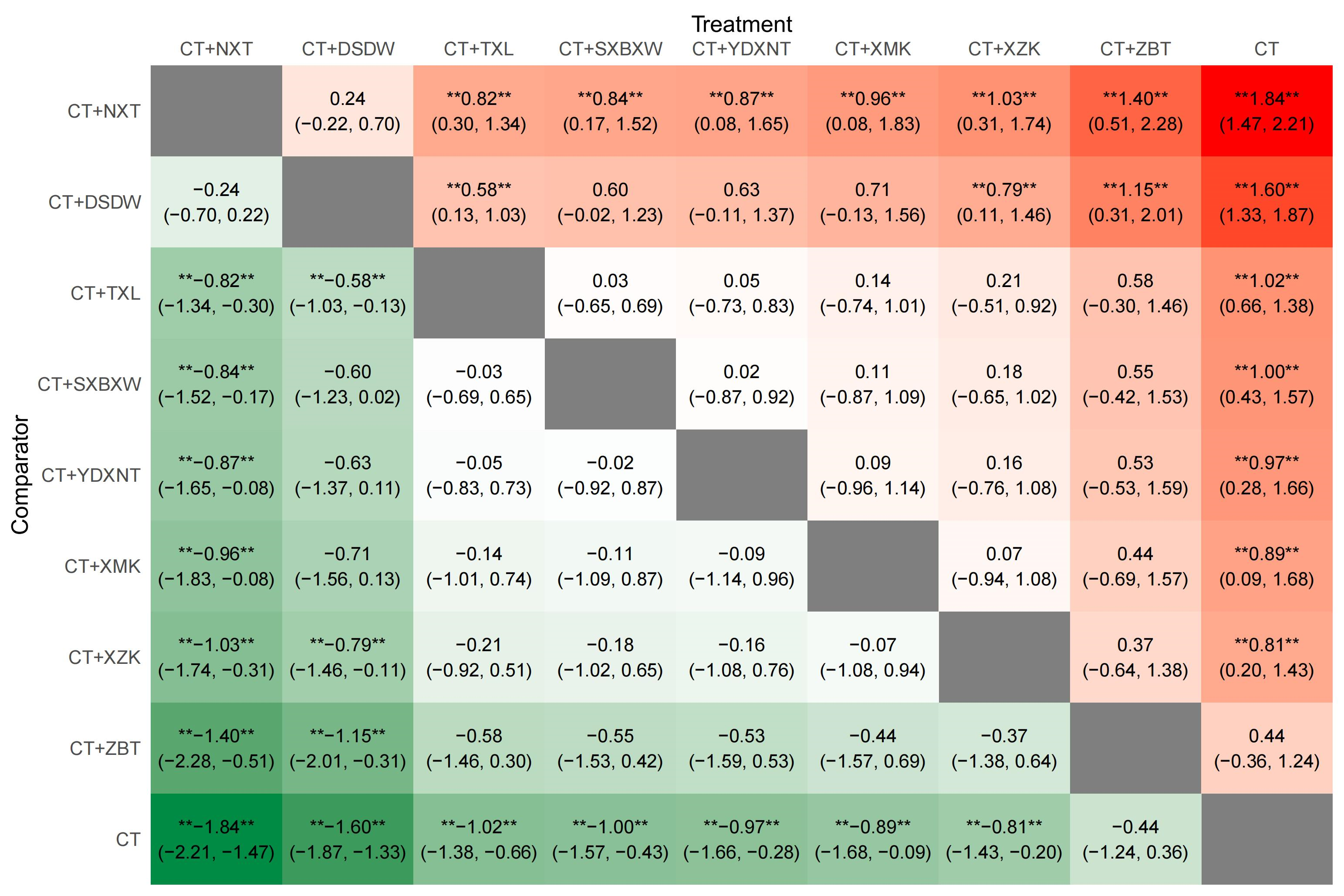

Supplement: Supplementary file 21 — Additional file 21: Figure S25. Network Meta-analysis heat map of TC [MD(95%CI)]. [file 13020_2023_866_MOESM21_ESM.png]

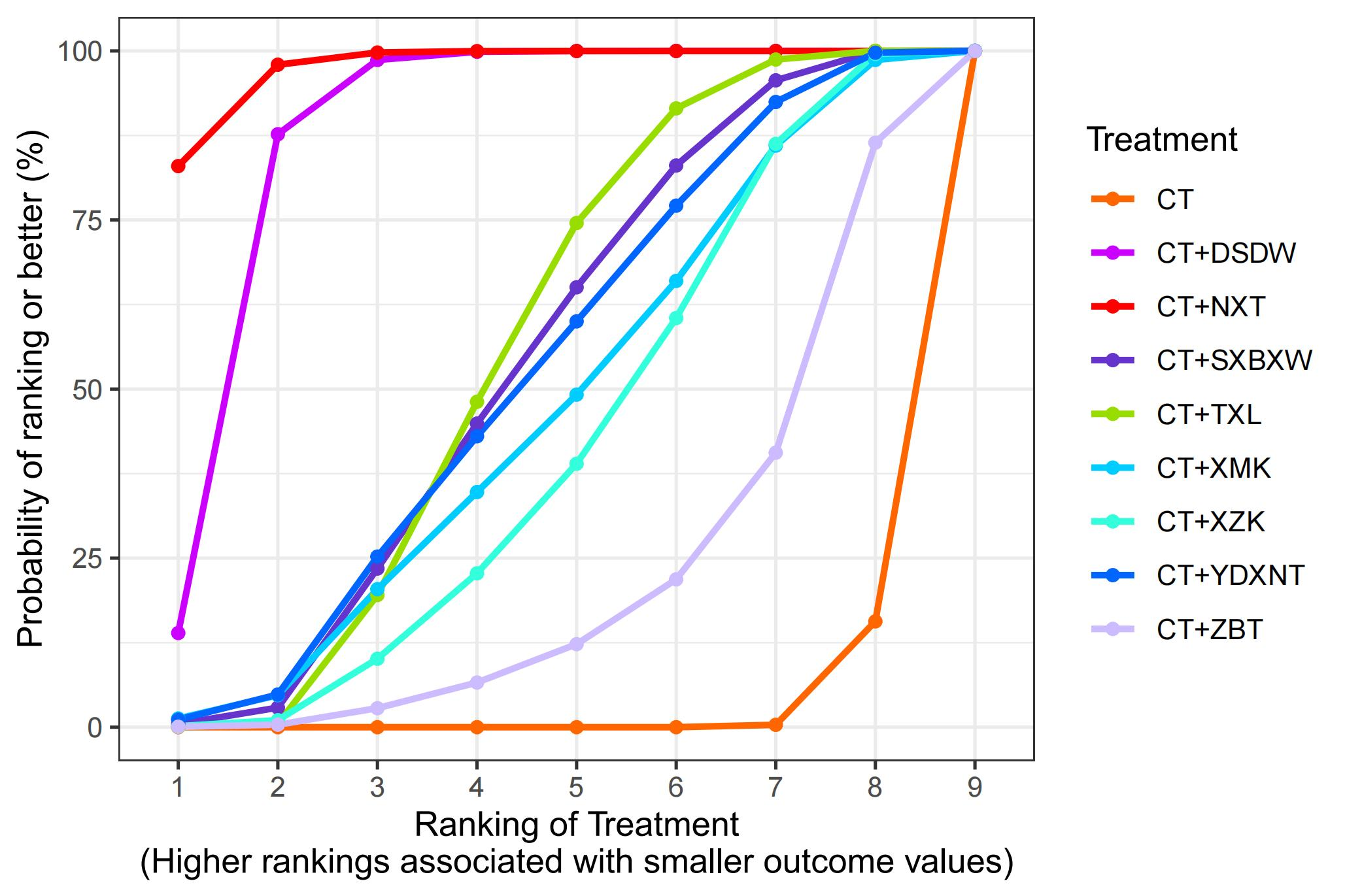

Supplement: Supplementary file 22 — Additional file 22: Figure S26. Probability ranking curves of the degree of TC reduction [MD(95%CI)]. [file 13020_2023_866_MOESM22_ESM.png]

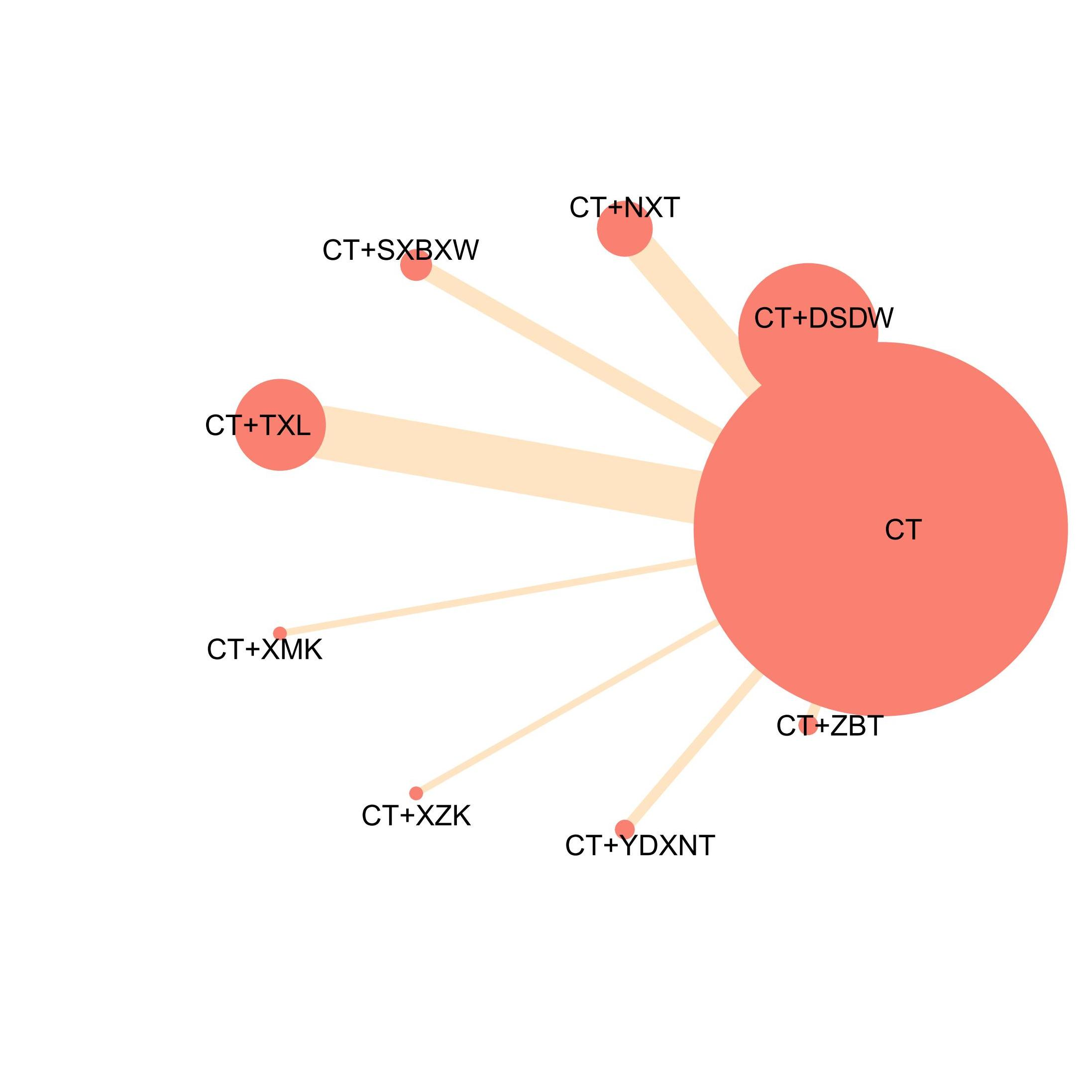

Supplement: Supplementary file 23 — Additional file 23: Figure S27. Evidence network map of total clinical effectiveness rate. [file 13020_2023_866_MOESM23_ESM.png]

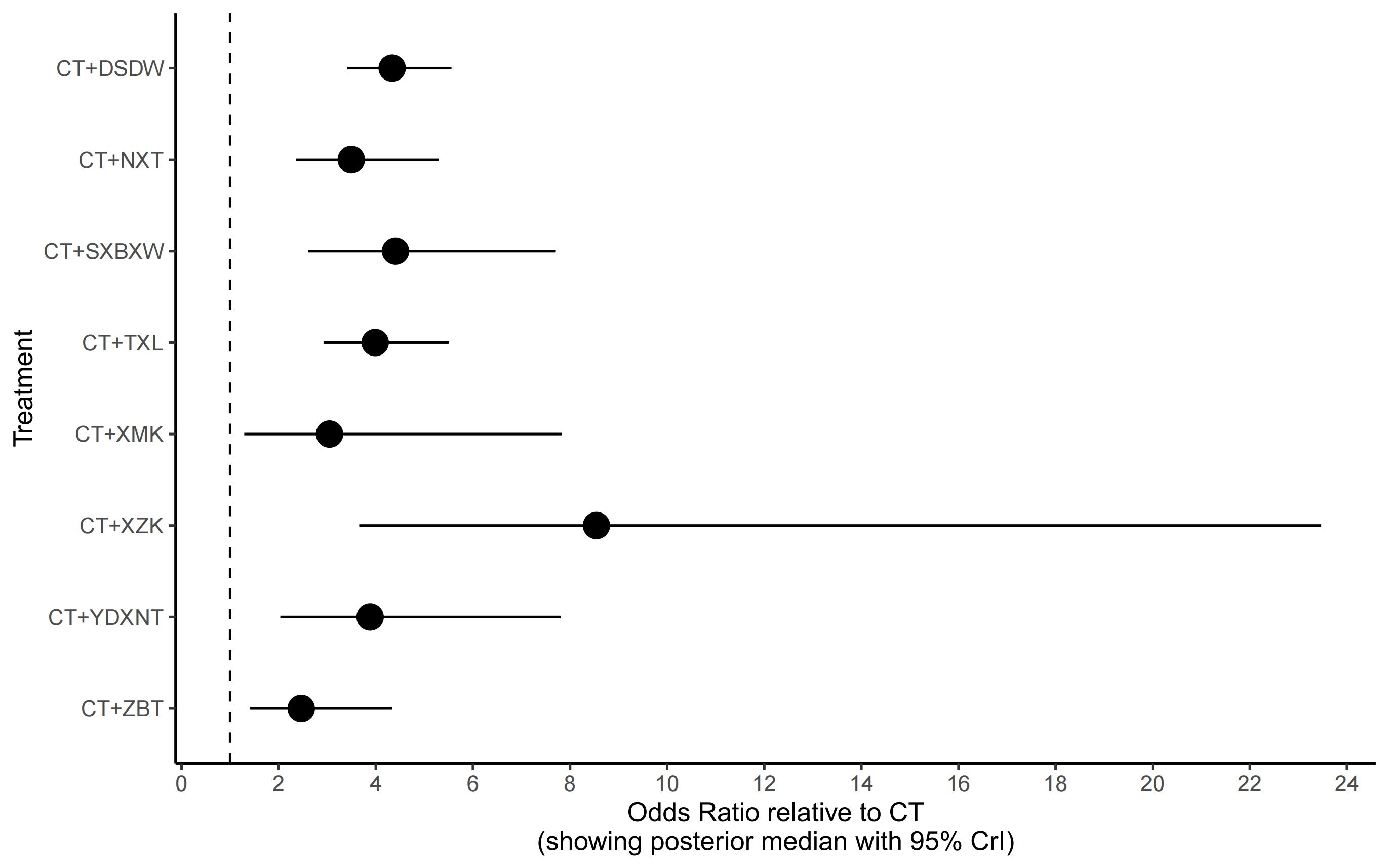

Supplement: Supplementary file 24 — Additional file 24: Figure S28. Direct comparison forest map of total clinical effectiveness rate [MD(95%CI)]. [file 13020_2023_866_MOESM24_ESM.png]

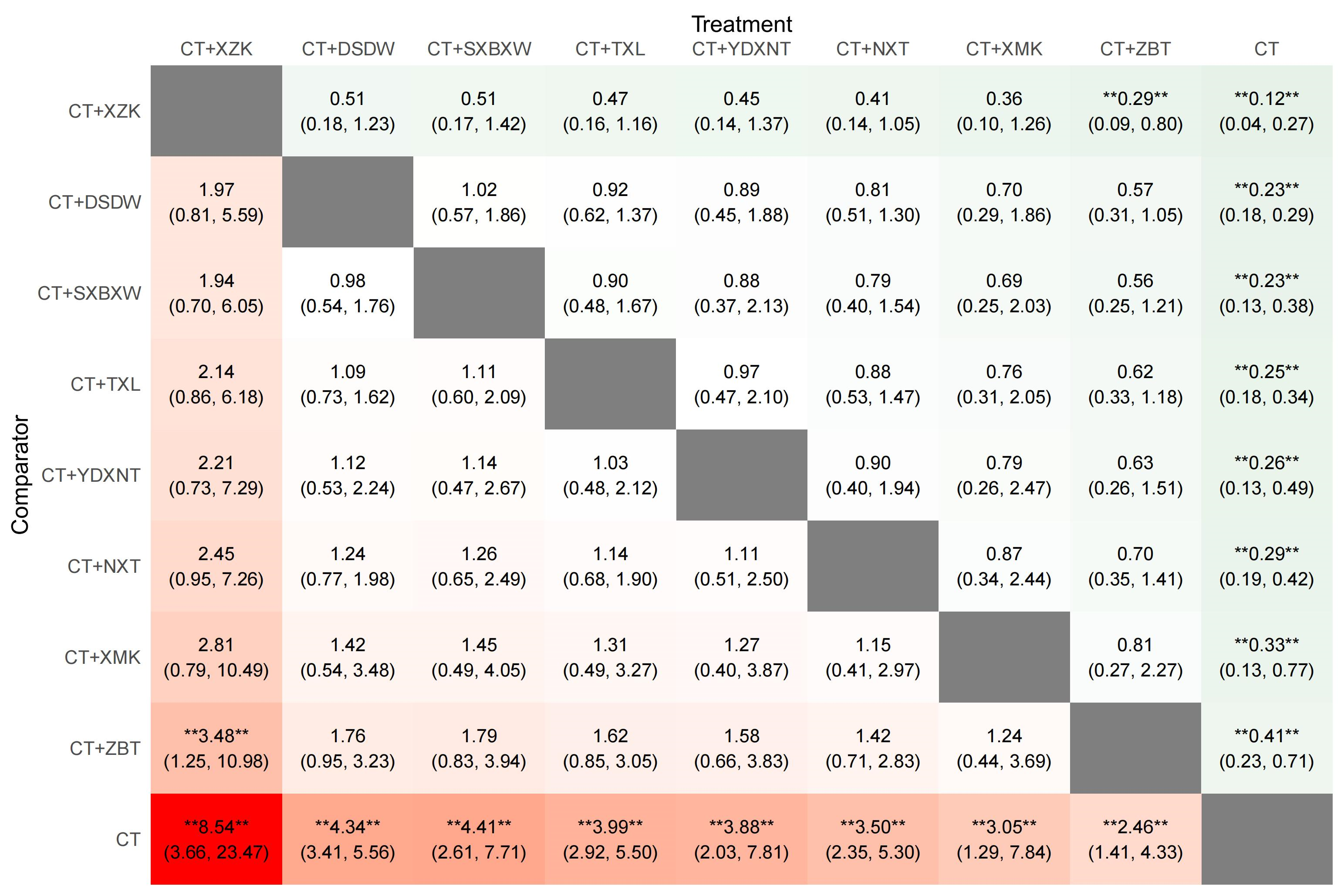

Supplement: Supplementary file 25 — Additional file 25: Figure S29. Network Meta-analysis heat map of total clinical effectiveness rate [MD(95%CI)]. [file 13020_2023_866_MOESM25_ESM.png]

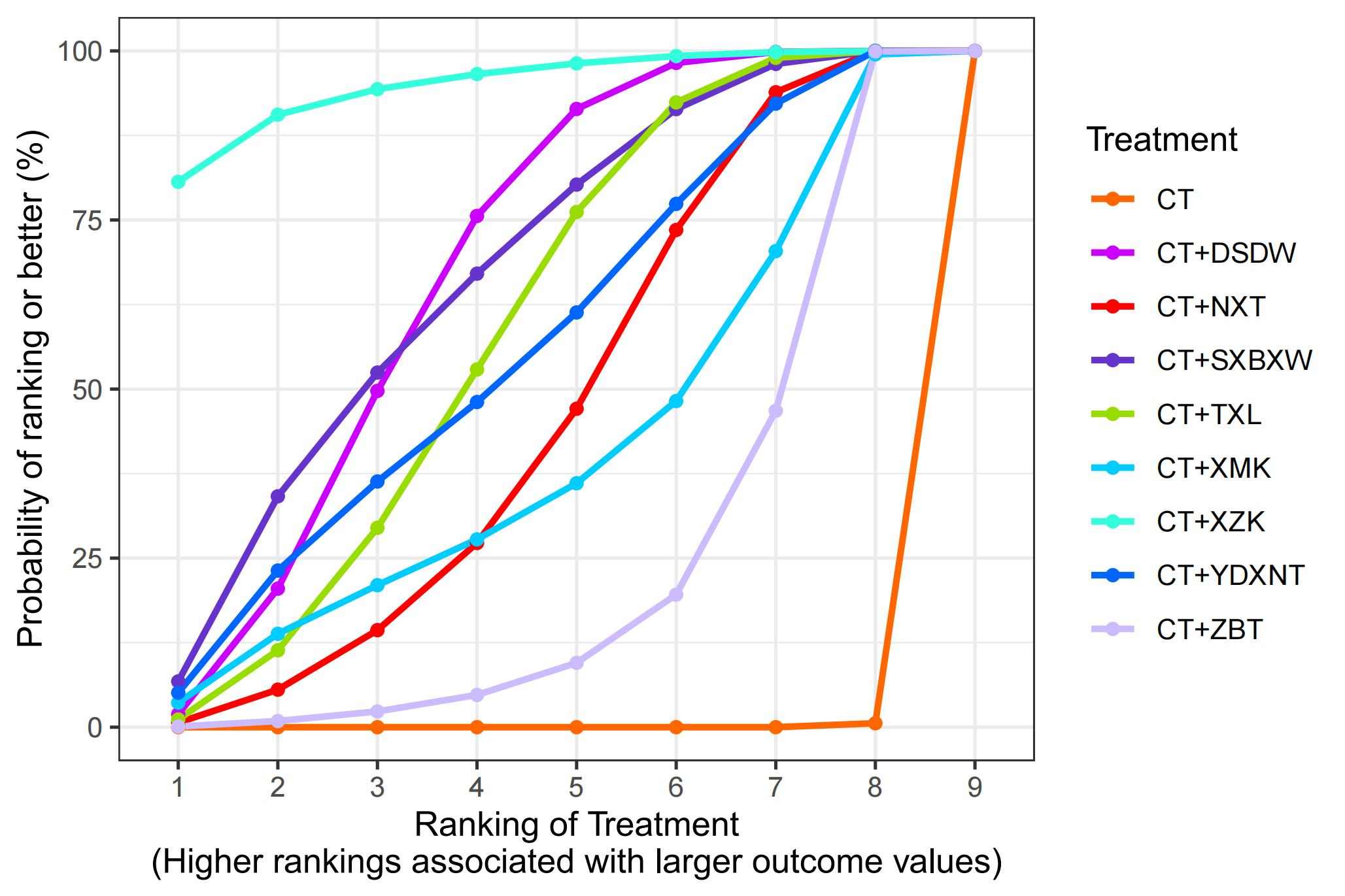

Supplement: Supplementary file 26 — Additional file 26: Figure S30. Probability ranking curves of the degree of improvement of total clinical effectiveness rate [MD (95%CI)]. [file 13020_2023_866_MOESM26_ESM.png]

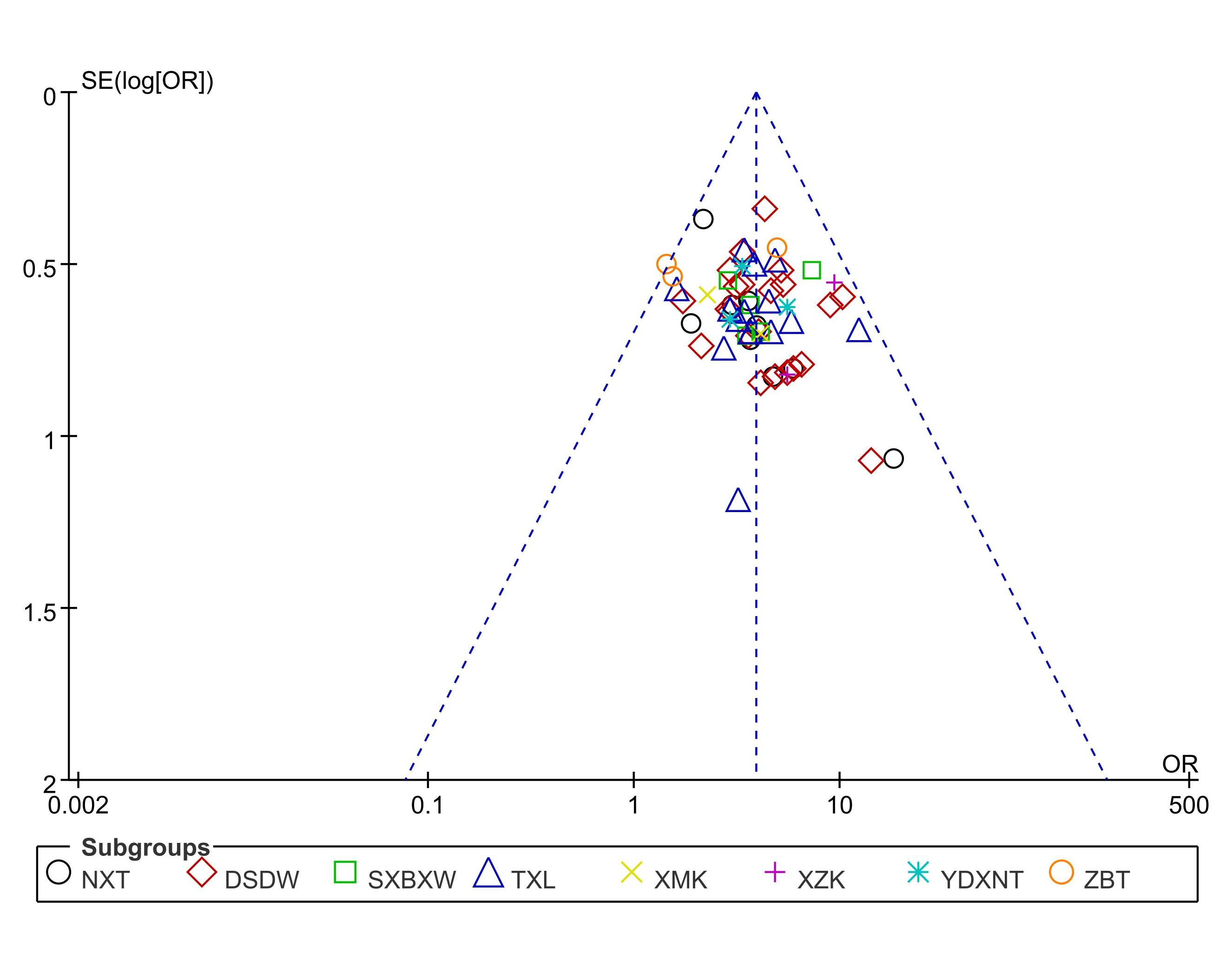

Supplement: Supplementary file 29 — Additional file 29: Figure S31. Funnel plot of the risk of publication bias. [file 13020_2023_866_MOESM29_ESM.png]
