# Supplementary material for: Efficacy and safety of oral Chinese patent medicines in the treatment of coronary heart disease combined with hyperlipidemia: a systematic review and network meta-analysis of 78 trials
Source: Chin Med. 2023 Dec 13;18:162. doi: 10.1186/s13020-023-00866-x (PMC10717272; doi:10.1186/s13020-023-00866-x)
Supplement: Supplementary file 27 — Additional file 27: Table S4. Adverse reaction statistics table. [file 13020_2023_866_MOESM27_ESM.docx]

**Table S4** Adverse reaction statistics table.

| Studay | Intervention | | Sample size (T/C) | Dizziness  (T/C) | Nausea  (T/C) | Headache  (T/C) | Diarrhea  (T/C) | Abdominal distention (T/C) | Skin rash (T/C) | Insomnia  (T/C) |
| --- | --- | --- | --- | --- | --- | --- | --- | --- | --- | --- |
|  | T | C |  |  |  |  |  |  |  |  |
| Li and Yan (2008) | CT+NXT | CT | 32/32 | - | - | - | - | - | - | - |
| Hao et al. (2016) | CT+NXT | CT | 95/95 | - | - | - | - | - | - | - |
| Wu (2011) | CT+NXT | CT | 50/50 | 1/0 | 1/0 | - | - | - | - | - |
| Deng (2015) | CT+DSDW | CT | 49/48 | - | - | - | - | - | - | - |
| Li (2019) | CT+DSDW | CT | 88/78 | - | - | 1/0 | 1/0 | - | - | - |
| Gao et al. (2007) | CT+NXT | CT | 45/45 | - | - | - | 1/0 | 1/0 | - | - |
| Gao et al. (2011) | CT+NXT | CT | 64/62 | 1/0 | 1/0 | - | - | - | - | - |
| Li (2016) | CT+NXT | CT | 40/40 | - | - | - | 1/0 | - | - | - |
| Lu (2014) | CT+NXT | CT | 44/44 | - | - | - | - | - | - | - |
| Zhang et al. (2014) | CT+NXT | CT | 56/60 | 1/0 | 1/0 | - | - | - | - | - |
| Hu (2021) | CT+SXBX | CT | 43/43 | 1/1 | - | 0/1 | - | - | 2/2 | - |
| Cai et al. (2015) | CT+XMK | CT | 48/48 | - | - | - | 3/0 | 2/4 | - | - |
| Yang (2014) | CT+XMK | CT | 35/35 | - | - | - | - | - | - | - |
| Zhang and Wei (2006) | CT+TXL | CT | 30/30 | - | - | - | - | 1/0 | - | - |
| Li (2004) | CT+TXL | CT | 36/24 | - | 1/0 | - | - | - | - | - |
| Zhang and Zhang (2005) | CT+TXL | CT | 60/60 | - | - | - | - | - | - | - |
| Chen (2004) | CT+TXL | CT | 30/30 | - | - | - | 2/1 | - | - | - |
| Qu (2015) | CT+XZK | CT | 100/100 | - | - | - | - | - | - | - |
| Zhao et al. (2008) | CT+XZK | CT | 30/30 | - | - | - | - | - | - | - |
| Huang et al. (2009) | CT+XZK | CT | 43/42 | - | - | - | - | - | - | - |
| He et al. (2008b) | CT+YDXNT | CT | 35/33 | - | 2/0 | - | - | - | - | - |
| Chen (2013) | CT+ZBT | CT | 70/35 | - | - | 1/2 | 1/2 | - | - | 2/1 |
| Shi (2008) | CT+ZBT | CT | 35/35 | - | - | - | - | - | - | - |
| Li et al. (2009) | CT+XZK | CT | 60/60 | - | - | - | - | - | - | - |
