# Supplementary material for: Efficacy and safety of oral Chinese patent medicines in the treatment of coronary heart disease combined with hyperlipidemia: a systematic review and network meta-analysis of 78 trials
Source: Chin Med. 2023 Dec 13;18:162. doi: 10.1186/s13020-023-00866-x (PMC10717272; doi:10.1186/s13020-023-00866-x)
Supplement: Supplementary file 28 — Additional file 28: Table S5. Table of subgroup analysis. [file 13020_2023_866_MOESM28_ESM.docx]

**Table S5** Table of subgroup analysis.

| outcome indicator | Basis for grouping | Number of studies | Sorting based on SUCRA values |
| --- | --- | --- | --- |
| CI | Simvastatin | 4 | NXT + CT > DSDW + CT > SXBXW + CT > CT |
|  | Atorvastatin | 8 | TXL + CT > DSDW + CT > CT |
|  | Ravastatin | 5 | DSDW + CT > CT |
|  | nitrates | 1 | TXL + CT > CT |
| CO | Simvastatin | 4 | NXT + CT > DSDW + CT > SXBXW + CT > CT |
|  | Atorvastatin | 8 | TXL + CT > DSDW + CT > CT |
|  | Ravastatin | 6 | DSDW + CT > CT |
|  | nitrates | 1 | TXL + CT > DSDW + CT > CT |
| HDL-C | Simvastatin | 5 | NXT + CT > SXBXW + CT > DSDW + CT > XMK + CT > CT |
|  | Atorvastatin | 15 | TXL + CT > SXBXW + CT > NXT + CT > DSDW + CT > XZK + CT > ZBT + CT > CT |
|  | Ravastatin | 8 | TXL + CT > DSDW + CT > YDXNT + CT > ZBT+ CT > CT |
|  | nitrates | 14 | NXT + CT > TXL + CT > SXBXW + CT > DSDW + CT > XZK + CT > XMK + CT > YDXNT + CT > CT |
|  | Lovastatin | 2 | TXL + CT > CT |
|  | Pivastatin | 1 | XZK + CT > CT |
|  | Trimetazidine | 1 | TXL + CT > CT |
| TG | Simvastatin | 10 | NXT + CT > SXBXW + CT > DSDW + CT > XMK + CT > CT |
|  | Atorvastatin | 16 | NXT + CT > TXL + CT > SXBXW + CT > DSDW + CT > XZK + CT > ZBT + CT > CT |
|  | Ravastatin | 13 | TXL + CT > DSDW + CT > SXBSW + CT > YDXNT + CT > ZBT + CT > CT |
|  | nitrates | 16 | NXT + CT > TXL + CT > SXBXW + CT > DSDW + CT > XMK + CT > XZK + CT > YDXNT + CT > CT |
|  | Lovastatin | 2 | TXL + CT > CT |
|  | Trimetazidine | 2 | TXL + CT > CT |
| LDL-C | Simvastatin | 7 | NXT + CT > DSDW + CT > XMK + CT > SXBXW + CT > CT |
|  | Atorvastatin | 15 | NXT + CT > DSDW + CT > TXL + CT > ZBT + CT > SXBXW + CT > CT |
|  | Ravastatin | 16 | TXL + CT > DSDW + CT > ZBT + CT > YDXNT + CT > SXBXW + CT > CT |
|  | nitrates | 14 | NXT + CT > DSDW + CT > TXL + CT > XZK + CT > XMK + CT > YDXNT + CT > SXBXW + CT > CT |
|  | Lovastatin | 4 | TXL + CT > DSDW + CT > CT |
|  | Pivastatin | 1 | XZK + CT > CT |
|  | Trimetazidine | 2 | TXL + CT > CT |
| TC | Simvastatin | 10 | NXT + CT > DSDW + CT > SXBXW + CT > XMK + CT > CT |
|  | Atorvastatin | 16 | NXT + CT > DSDW + CT > SXBXW + CT > TXL + CT > XZK + CT > ZBT + CT > CT |
|  | Ravastatin | 16 | DSDW + CT > TXL + CT > YDXNT + CT > SXBXW + CT > ZBT + CT > CT |
|  | nitrates | 16 | NXT + CT > DSDW + CT > TXL + CT > SXBXW + CT > YDXNT + CT > XMK + CT > XZK + CT > CT |
|  | Lovastatin | 4 | DSDW + CT > TXL + CT > CT |
|  | Pivastatin | 1 | XZK + CT > CT |
|  | Trimetazidine | 2 | TXL + CT > CT |
| Total clinical effectiveness rate | Simvastatin | 9 | DSDW + CT > SXBXW + CT > NXT + CT > XMK + CT > CT |
|  | Atorvastatin | 14 | XZK + CT > SXBXW + CT > DSDW + CT > TXL + CT > NXT + CT > ZBT + CT > CT |
|  | Ravastatin | 14 | DSDW + CT > SXBXW + CT > TXL + CT > YDXNT + CT > ZBT + CT > CT |
|  | nitrates | 12 | DSDW + CT > SXBXW + CT > TXL + CT > YDXNT + CT > XMK + CT > NXT + CT > CT |
|  | Lovastatin | 4 | DSDW + CT > TXL + CT > CT |
|  | Pivastatin | 1 | XZK + CT > CT |
|  | Trimetazidine | 2 | TXL + CT > CT |
