# Supplementary material for: Efficacy and safety of oral Chinese patent medicines in the treatment of coronary heart disease combined with hyperlipidemia: a systematic review and network meta-analysis of 78 trials
Source: Chin Med. 2023 Dec 13;18:162. doi: 10.1186/s13020-023-00866-x (PMC10717272; doi:10.1186/s13020-023-00866-x)
Supplement: Supplementary file 30 — Additional file 30. Abbreviations. [file 13020_2023_866_MOESM30_ESM.docx]

**Abbreviations**

RCTs randomized controlled trials

CHD coronary heart disease

HLP hyperlipidemia

CT conventional treatment

CI cardiac index

CO cardiac output

LDL-C low-density lipoprotein cholesterol

TC total cholesterol

HDL-C high-density lipoprotein cholesterol

TG triglyceride

AEs Adverse Events

T treatment group

C control group

DSDW Compound Danshen Dripping Pill

NXT Naoxintong Capsule

SXBXW Shexiangbaoxin Pill

XMK Songling Xuemaikang Capsule

TXL Tongxinluo Capsule

XZK Xuezhikang Capsule

YDXNT Yindan Xinnaotong Capsule

ZBT Zhibitai Capsule

WHO World Health Organization

JAGS Just Another Gibbs Sampler

DIC deviance information criterion

SUCRA surface under the cumulative ranking

MD mean deviation

CNKI China National Knowledge Infrastructure

VIP China Science and Technology Journal Database

KEGG Kyoto Encyclopedia of Genes and Genomes

PAI-1 plasminogen activator inhibitor-1

PPARγ peroxisome proliferator-activated receptor γ

eNOS endothelial Nitric Oxide Synthases

JNK c-Jun N-terminal kinase
