# Supplementary material for: Efficacy and safety of oral Chinese patent medicines in the treatment of coronary heart disease combined with hyperlipidemia: a systematic review and network meta-analysis of 78 trials
Source: Chin Med. 2023 Dec 13;18:162. doi: 10.1186/s13020-023-00866-x (PMC10717272; doi:10.1186/s13020-023-00866-x)
Supplement: Supplementary file 31 — Additional file 31. Drug approval number. [file 13020_2023_866_MOESM31_ESM.docx]

**Drug approval number**

Tongxinluo Capsule: National Drug Certification Z19980015

Naoxintong Capsule: National Drug Certification Z20025001

Compound Danshen Dripping Pill: National Drug Certification Z10950111

Shexiangbaoxin Pill: National Drug Certification Z31020068

Songling Xuemaikang Capsule: National Drug Certification Z10960023

Xuezhikang Capsule: National Drug Certification Z10950029

Yindan Xinnaotong Capsule: National Drug Certification Z20027144

Zhibitai Capsule: National Drug Certification Z51022196
